# Targeting interleukin-6 as a treatment approach for peritoneal carcinomatosis

**DOI:** 10.1186/s12967-024-05205-8

**Published:** 2024-04-30

**Authors:** Neda Dadgar, Christopher Sherry, Jenna Zimmerman, Hyun Park, Catherine Lewis, Albert Donnenberg, Ali H. Zaidi, Yong Fan, Kunhong Xiao, David Bartlett, Vera Donnenberg, Patrick L. Wagner

**Affiliations:** 1 Translational Hematology & Oncology Research, Enterprise Cancer Institute, Cleveland Clinic, Cleveland, OH 44106 USA; 2 Allegheny Health Network Cancer Institute, 314 E. North Ave, Pittsburgh, PA 15212 USA; 3 Center for Proteomics & Artificial Intelligence, Center for Clinical Mass Spectrometry, Allegheny Health Network Cancer Institute, Pittsburgh, PA 15224 USA; 4 University of Pittsburgh School of MedicineDepartment of Cardiothoracic SurgeryUPMC Hillman Cancer Center Wagner, Patrick; Allegheny Health Network Cancer Institute, Pittsburgh, USA

**Keywords:** IL-6, Peritoneal carcinomatosis, Targeted therapy, Tumor immune microenvironment

## Abstract

Peritoneal carcinomatosis (PC) is a complex manifestation of abdominal cancers, with a poor prognosis and limited treatment options. Recent work identifying high concentrations of the cytokine interleukin-6 (IL-6) and its soluble receptor (sIL-6-Rα) in the peritoneal cavity of patients with PC has highlighted this pathway as an emerging potential therapeutic target. This review article provides a comprehensive overview of the current understanding of the potential role of IL-6 in the development and progression of PC. We discuss mechansims by which the IL-6 pathway may contribute to peritoneal tumor dissemination, mesothelial adhesion and invasion, stromal invasion and proliferation, and immune response modulation. Finally, we review the prospects for targeting the IL-6 pathway in the treatment of PC, focusing on common sites of origin, including ovarian, gastric, pancreatic, colorectal and appendiceal cancer, and mesothelioma.

**Supplementary Information:**

The online version contains supplementary material available at 10.1186/s12967-024-05205-8.

## Background

Peritoneal carcinomatosis (PC) is a late manifestation of abdominopelvic malignancies, commonly associated with gastrointestinal and gynecological cancers, such as ovarian, colorectal, and pancreatic cancer [1, 2]. Marked by poor prognosis and limited treatment options, PC causes severe symptoms related to intestinal obstruction and nutritional compromise. Treatment is generally considered palliative, as disease recurrence is the rule rather than the exception. Coventional treatment modalities include systemic chemotherapy as well as cytoreductive surgery, both of which are plagued with limited efficacy and significant toxicity. Intraperitoneal chemotherapy holds theoretical advantages in selected patients, although there is mixed evidence regarding its contribution to oncologic outcome in most cancer types [3–6].

Given the limitations of conventional modalities in treating PC, there is a pressing need to develop novel and innovative strategies for this metastatic pattern. Immunotherapy presents an obvious candidate approach, to which the peritoneal space may be well suited as a distinct immune environment segrated from the systemic circulation. Regional immunotherapy directed toward the peritoneal cavity has been explored as a an alternative strategy to systemic therapy and complementaty to cytoreductive surgery. Such strategies include the use of cytokines, monoclonal antibodies, immune checkpoints, vaccines, viral oncotherapy and adoptive cellular therapeutics [7–12]. In spite of a strong rationale, none of these therapeutic modalities has gained footing in routine clinical management for PC patients to date.

In order to improve the prospects for success in using regional therapy to treat PC, we and others have recently sought to better define the immune milieu of the peritoneal environment [13, 14]. It is hoped that by further characterizing the baseline physiologic status of the peritoneal environment and the changes that occur during carcinomatosis, we will identify potential targets and novel combination strategies to address this metastasis-prone anatomic compartment [15]. Based on our discovery of high concentration of both IL-6 and its cognate soluble receptor in the peritoneal cavity of PC patients [16, 17], we have identified the IL-6 signalling pathway as one such potential target [18–21]. Recent studies have shed light on the potential association between IL-6 and the development of PC, as well as the role of IL-6 in peritoneal tumour dissemination, mesothelial adhesion and invasion, stromal invasion and proliferation, and immune response modulation [22–25]. Furthermore, the use of immunotherapy in the treatment of PC has shown significant promise in preclinical and clinical studies [26, 27].

In this review article, we will provide an overview of the current understanding of the integral role of IL-6 in the development and progression of PC and discuss the potential therapeutic implications in cancers that commonly cause PC, including ovarian cancer, gastric cancer, colorectal cancer and ovarian cancer. By reviewing the current state of knowledge on the impact of the IL-6 axis in PC biology, we aim to reveal or expose opprotunities to target this pathway intra-peritoneally in patients with PC.

### The intricacies of IL-6 biology: from signaling pathways to therapeutic potential

IL-6 is a pleiotropic master cytokine with normal physiologic functions, as well as a central role in a number of pathologic states including inflammation, infectious disease, autoimmune disorders and cancer [28, 29]. IL-6 serves as a diagnostic and prognostic biomarker in several diseases, aiding in detection, monitoring, and predicting treatment response [30]. The diverse effects of IL-6 on cellular signaling and immune responses make it an attractive target for developing effective therapies for these pathologic conditions [31]. The downstream signaling pathways activated by IL-6 are multifaceted and context-dependent. Classical signaling involves the binding of IL-6 to its membrane-bound receptor, the IL-6Rα-gp130 complex, leading to activation of the JAK/STAT3 and MAPK pathways and subsequent downstream gene transcription via NF-κB [32, 33]. The membrane-bound IL-6R complex is composed of two subunits, one of which (IL-6Rα) is specific to IL-6 and the other, gp130, is shared among all type I cytokine family members [34, 35]. Trans signalling occurs when IL-6 binds to the soluble form of IL-6Rα (sIL-6Rα) [36]. Because gp130 expression is far more widespread among a variety of cell types, trans signaling can affect a much broader set of target tissues, as may be the case in the peritoneal cavity, since both IL-6 and sIL-6Rα are present at extremely high concentration in patients with PC [21].

In the context of inflammation [37], infectious disease, or autoimmunity, IL-6 signaling is a key stimulus for innate immune responses and, when dysregulated, is implicated in the pathogenesis of autoimmune disorders [38, 39]. In cancer, IL-6 signalling promotes tumor growth, metastasis [40, 41] and immune evasion [42]. The central position of IL-6 at the intersection between inflammation, innate immune response, and cell proliferation offers significant opportunities for therapeutic intervention [43]. Monoclonal antibodies directed against IL-6 or its receptor, as well as small molecule inhibitors of the IL-6 signaling pathway are being investigated as potential therapeutic modalities [44] (Table 1). Combining antagonists of the IL-6 pathway with other treatment approaches holds promise for enhancing efficacy and improving patient outcomes [45], and ongoing clinical trials are evaluating the safety and efficacy of IL-6 inhibition in cancer patients [44]. However, challenges such as treatment resistance and patient heterogeneity need to be addressed in order to further optimize IL-6-based therapeutics.

**Table 1:** IL-6 Pathway inhibitors in clinical use or investigation

Drug	Mechanism of Action	Stage of Development	Company	Ref.
Tocilizumab	Monoclonal antibody interleukin-6 (IL-6R) receptor	Approved	Roche/Genentech	[252, 253]
Sarilumab	Monoclonal antibody targeting nterleukin (IL)-6 receptors (sIL-6Rα and mIL-6Rα)	Approved	Sanofi/Regeneron	[254–256]
Vobarilizumab	Monoclonal antibody interleukin-6 (IL-6) receptor	Phase II/III	Ablynx	[257]
Satralizumab	Monoclonal antibody interleukin-6 (IL-6) receptor	Phase III	Roche	[258]
NI-1201	Monoclonal antibody interleukin-6 (IL-6) receptor	Preclinical	Tiziana	[259]
Olokizumab	Monoclonal antibody interleukin-6 (IL-6) receptor	Phase III	R-Pharm	[260]
Ziltivekimab	Monoclonal antibody directed against the IL-6 ligand	Phase II	Novo Nordisk	[261]
Canakinumab	Monoclonal antibody targeting IL-1β	Phase II	Novartis	[262]
GSK3196165	Small molecule inhibitor of IL-6	Phase II	GlaxoSmithKline	[263]
Atlizumab(MRA)	Recombinant human anti-interleukin-6 (anti-IL-6) receptor monoclonal antibody	phase I/II	Chugai Pharmaceutical	[264–266]
Elsilimomab(B-E8)	Monoclonal antibodies against interleukin-6	preclinical	Diaclone	[267]
Levilimab(BCD-089)	IL-6 receptor inhibitor	phase III	phase III	[268]
Olamkicept (sgp130Fc)	IL-6 receptor antagonist	Phase II	Olam Labs	[269, 270]
Clazakizumab	Anti–IL-6 Antibody	Phase II/III	CSL Behring/Vitaeris/ Novartis	[271, 272]
Siltuximab	Monoclonal antibody targeting IL-6	Approved	EUSA Pharma	[273]
Sirukumab	Monoclonal antibody targeting IL-6	Phase III	Janssen	[274, 275]
Leronlimab	Monoclonal antibody targeting CCR5	Phase III	CytoDyn	[276]
Emapalumab	Monoclonal antibody targeting IFN-γ	Phase II/III	Swedish Orphan Biovitrum (Sobi)	[277]

### IL-6 in peritoneal pathophysiology

**Fig. 1:**
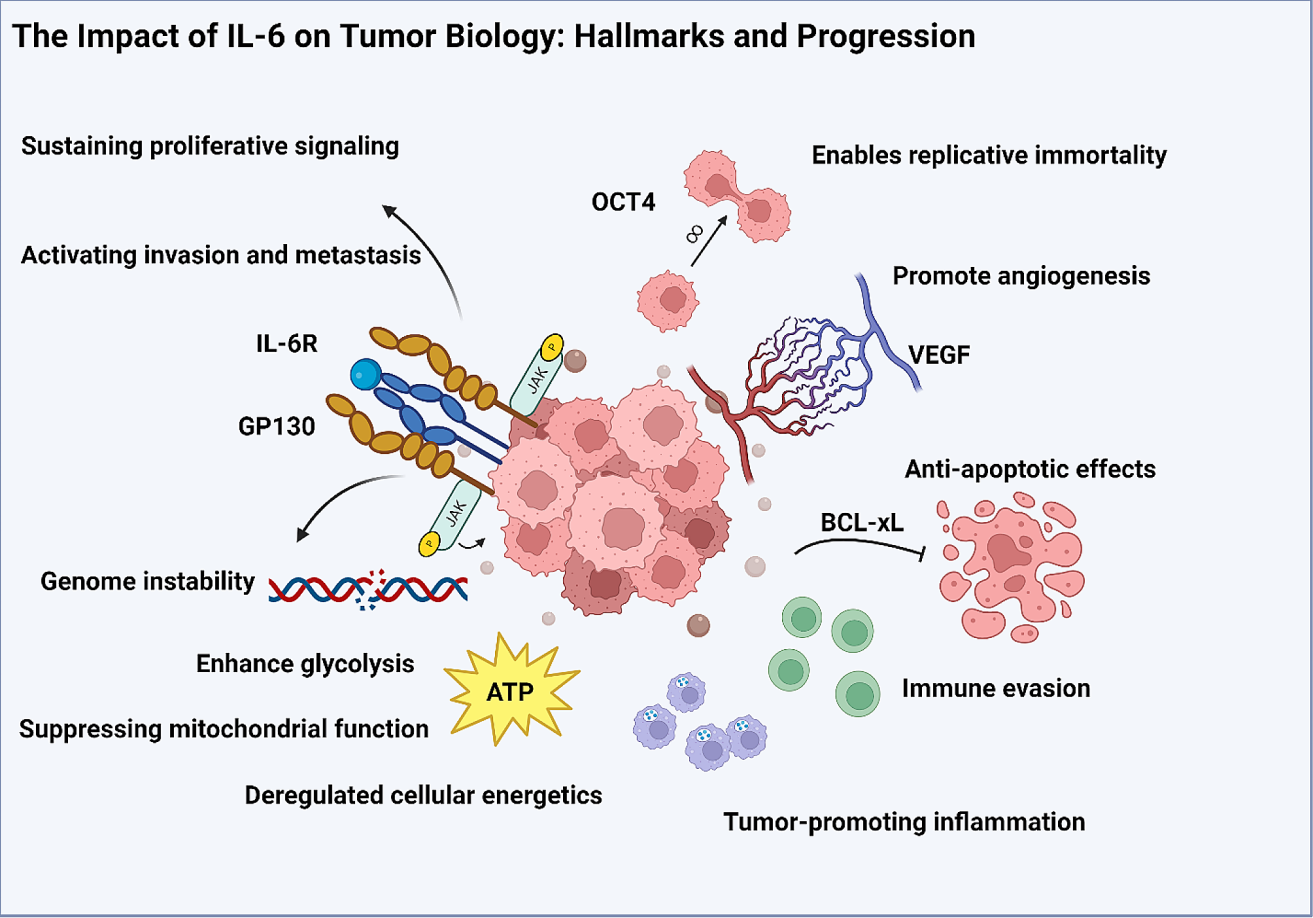
Overview of interleukin-6 (IL-6) impact on key hallmarks of cancer. IL-6 drives sustained proliferative signaling, activation of invasion and metastasis, and contributes to genome instability and immune evasion within the tumor microenvironment. Additionally, IL-6 enables replicative immortality, promotes angiogenesis, exerts anti-apoptotic effects, and influences cellular energetics, collectively driving cancer progression and highlighting its pivotal role in tumor biology. Created with BioRender.com

The peritoneal cavity serves as a crucial site for immune responses and plays a significant role in maintaining homeostasis within the body [46]. Within the peritoneal cavity, IL-6 is produced by several cell types. Mesothelial cells, a monolayer of specialized cells lining the peritoneal cavity, are known to secrete IL-6 in response to inflammatory signals [47, 48]. Resident macrophages within the peritoneal cavity have also been shown to produce IL-6 in response to various stimuli, presumably as an early response mechanism to breaches of the intestinal tissue due to injury or by invading pathogens [49]. Fibroblasts within the peritoneal cavity are also capable of producing IL-6 upon activation [50, 51], as are neutrophils in the setting of early response to inflammation or infection [52]. T lymphocytes, including CD4 + and CD8 + T cells, infiltrate the peritoneal cavity during immune responses and can produce IL-6, either directly or indirectly, depending on the context and activation status [53, 54]. Other infiltrating immune cells, such as activated B cells [55, 56], natural killer (NK) cells, and dendritic cells, have been implicated in IL-6 production within the peritoneal cavity, albeit to a lesser extent [57]. Within these cells, signaling pathways, such as NF-κB, STAT3, AP-1, MAPK, and PI3K-Akt, mediate IL-6 production and downstream effects [58–60]. While the precise molecular mechanisms governing IL-6 production by different peritoneal cell populations remain poorly characterized, a better understanding of these processes could potentially facilitate the development of therapies aimed at modulating IL-6 production and its downstream effects fig. 1

IL-6 exerts diverse effects in different peritoneal diseases. As a central cytokine, its effects have been documented to promote inflammatory processes, immune responses, and tumor biology, and to contribute to the pathogenesis and progression of peritonitis, peritoneal dialysis-related complications, ascites, peritoneal fibrosis, and peritoneal cancer, as referenced below. While IL-6 exhibits distinct roles in various conditions, common effects are shared among them. IL-6 promotes the recruitment and activation of immune cells [61], resulting in an inflammatory response within the peritoneal cavity. This inflammatory environment can contribute to tissue damage, fibrosis, and disease progression [62, 63]. In peritonitis, IL-6-induced immune responses contribute to leukocyte recruitment and activation [64]. However, excessive or dysregulated immune responses mediated by IL-6 can also contribute to the chronic inflammation and tissue damage seen in peritoneal dialysis-related complications and peritoneal fibrosis [65]. IL-6 stimulates the production of extracellular matrix components, such as collagen, leading to fibrotic tissue deposition. Excessive fibrosis can impair organ function and compromise peritoneal dialysis efficiency, and elevated levels of IL-6 have been associated with poor prognosis in peritoneal fibrosis [66, 67]. In peritoneal dialysis-associated peritonitis, inflammatory factors and fibrotic mediators reduce the secretion of decorin by peritoneal mesothelial cells (PMCs), causing excessive deposition of fibronectin secreted by PMCs and fibrosis [68–70]. Inhibition of IL-6 signaling, either through monoclonal antibodies or small molecule inhibitors, has shown promise in preclinical and clinical studies for the management of inflammation and fibrosis [43]. In endometriosis, the IL-6 pathway has long been implicated as a central driver of fibrotic pathology [71–73], with more recent studies identifying persistent activation of STAT3 via IL-6 trans-signaling as a driving mechanism, and highlighting IL-6 inhibition as a potential therapeutic intervention [74].

### The impact of IL-6 on tumor biology: hallmarks and progression

Circulating IL-6 has been defined as a prognostic marker in various cancer types, implying significance in tumor biology [75]. The classic and trans-signaling pathways of IL-6 provide insights into the intricate mechanisms by which IL-6 may contribute to cancer progression [76]. The prototypical hallmarks of cancer biology, as defined by Hanahan and Weinberg [77], encompass essential characteristics of tumor development and progression, including: sustaining proliferative signalling, evading growth supressors, avoid immune destruction, enabling replicative immortality, tumor-promoting inflammation, activating invasion and metastasis, inducing or accessing vasculature, genome instability and mutation, resisting cell death, and deregulating cellular metabolism. In this section, we highlight mechanisms whereby IL-6 biology can influence a number of these central processes in cancer biology.

IL-6 plays a crucial role in sustaining proliferative signaling in tumors that express IL-6Rα or gp130. IL-6Rα and gp130 expression have been characterized and correlated with prognosis in gastric, colorectal, and ovarian cancers, in which various downstream effector pathways, such as JAK-STAT, PI3K-Akt, and MAPK, led to mitogenic effects [78–80]. In a number of in vitro and cell line experiments, targeted inhibition of IL6-Rα abrogated these effects, consistent with a model in which IL-6 is a driver of tumorigenesis in tumor types often associated with PC [81–87]. IL-6 is centrally involved in activating EMT and promoting invasion and metastasis in tumors [41]. In colorectal cancer, for example, IL-6 signalling via STAT3 results in repression of a micro-RNA (miR-34a), that in turn results in increased expression of EMT signature genes associated with invasion and metastasis, such as vimentin, SNAIL, SLUG and ZEB1, with concommitant loss of E-cadherin [86].

While the direct relationship between IL-6 and genomic instability is not fully understood, IL-6-induced inflammation can contribute to this hallmark through various mechanisms. Firstly, IL-6 activates signaling pathways like JAK-STAT, which directly affects DNA repair processes, potentially leading to DNA damage accumulation and subsequent mutations [88]. Secondly, IL-6 promotes the recruitment and activation of immune cells, triggering the release of inflammatory mediators and reactive species that can cause oxidative DNA damage, increasing mutation risk [89]. Additionally, IL-6 modulates transcription factors involved in DNA repair and cell cycle regulation, disrupting DNA replication fidelity and increasing errors during synthesis [90]. Moreover, IL-6 influences the tumor microenvironment by promoting angiogenesis, EMT, and altering the extracellular matrix, creating a hypoxic and nutrient-deprived environment that further contributes to genomic instability and the selection of aggressive cancer cell populations [90, 91].

IL-6 has also been shown to promote angiogenesis via VEGF secretion by human peritoneal mesothelial cells, in the context of trans signalling (i.e., simultaneous exposure to IL-6 and sIL6-Rα) [92], as well as in the context of tumor stromal fibroblast-secreted IL-6 acting in an autocrine fashion to drive VEGF expression in colorectal cancers [93]. IL-6 facilitates replicative immortality in cancer cells by influencing the equilibrium between cancer stem cells and non-stem cells through regulation of OCT-4 gene expression [94]. IL-6 exerts anti-apoptotic effects, by potentiating expression of STAT3-driven genes such as BCL-xL and survivin [95]. BCL-xL is a member of the Bcl-2 family of anti-apoptotic genes which prevents mitochondrial release of cytochrome C, which cleaves caspases to initate the apoptotic cascade [96]. Survivin prevents apoptosis by direct inhibitory binding to caspases 3 and 7 [97]. IL-6 influences dysregulated cellular energetics, leading to enhancing glycolysis and suppressing mitochondrial function, enabling cancer cells to adapt to the demanding metabolic needs associated with rapid proliferation and survival with the necessary resources to support their growth and progression [98, 99].

As a master cytokine, IL-6 may play a role in immune evasion by tumors. Adaptive anti-tumor immunity, in general terms, relies heavily on cytotoxic responses to tumor neoantigens—a process largely driven by interferon-γ secretion by Th1 CD4 + T cells. IL-6 promotes differentiation of CD4 + T cells to a pro-inflammatory Th17 phenotype, and can even stimulate trans-differentation of Th1 cells into Th17 cells [100, 101]. In the context of cancer immunity, a Th17 polarity is maladaptive, driving the immune response toward chronic inflammation and away from adaptive cytotoxic immunity [102]. IL-6 and G-CSF from stromal or tumor sources can activate STAT3 signaling, resulting in an increase in suppressive immune effects of PMN-MDSCs by enhancing C/EBPβ expression and inhibiting IRF8 expression. Relatedly, IL-6 contributes to tumor-promoting inflammation in the tumor microenvironment by recruiting and activating MDSC’s, macrophages and tolerogenic dendritic cells [103]. The level of IL-6 in tumors has been linked to increased necrosis, proliferation, differentiation, and vascular invasion, while higher levels of IL-6 in the systemic circulation are associated with advanced T-stage, elevated CRP levels, and lower survival rates. As a result, IL-6 has been proposed as critical mediator of the connection between tumor necrosis, local and systemic inflammation, and patient outcomes in colorectal and other cancers [104].

### The potential association between interleukin-6 (IL-6) and the development of peritoneal carcinomatosis

Beyond its generic tumor-promoting effects, the IL-6 axis could make unique contributions specifically to PC as a unique metastatic pattern, irrespective of primary tumor site. A central role for IL-6 in promoting peritoneal carcinomatosis has been inferred from research consistently demonstrating elevated levels of IL-6 in the serum and ascites of patients with PC compared to those without it, in ovarian [105, 106], colorectal [107–109], gastric [110] and other cancers [17, 111–113]. In a recent study across multiple primary tumor types, we not only found elevated levels of IL-6 in the peritoneal fluid of patients with PC, but that the soluble receptor sIL6-Rα was present at exorbitant concentration regardless of disease state [21]. Thus, in the context of PC, the IL-6 cytokine and soluble receptor combination would create conditions ripe for trans signalling via the IL6 pathway to dominate peritoneal physiology during PC, and specifically to polarize the immune environment toward a counterproductive innate or chronic inflammatory response, allowing tumor cells to evade adaptive cytotoxic immunity [18, 114].

Bootsma et al. have recently described processes involved in PC as distinct from other routes of metastatic spread, such as lymph node or solid organ metastases [115]. These processes are briefly reviewed here to discuss the role of the IL-6 axis in each. PC can arise from metastatic cells through direct seeding of the peritoneal cavity by transmural tumor growth from an abdominopelvic organ, or by lymphatic or hematogenous routes. The nidus of metastatic growth could be either individual cancer cells, or clusters of cell aggregates that rely on adhesion molecules and fibrin deposition for cohesiveness. Once within the potential space of the peritoneum, tumor cells or clusters must adhere to and grow on the mesothelial surfaces. Several central themes emerge at this step, including the expression of cell adhesion molecules (integrins, ICAM, VCAM and others) on tumor and mesothelial cells, as well as features of EMT phenotype, promoting the etablishment of metastatic tumor implants complete with stroma and vascularization. The peritoneal cavity has an immune milieu that is quite distinct from that within the systemic circulation or indiviudal organs [116]. For example, the mucosal immune system of the gut is well known to promote tolerance of commensal organisms, whereas the peritoneal immune system appears polarized toward an immediate innate response to violation of luminal barriers in the context of infection or injury [70, 117]. Much work remains to decipher the influence of these underlying immune configurations on the processes at play in primary gastrointestinal tumor growth and on peritoneal progression in PC [115]. Below, we discuss the examples of the contribution of IL-6 on each of these elements of PC biology.

Direct proliferative effects of IL-6 on peritoneal metastatic cells have been documented in several tumor types relevant to PC, both in cell lines and in murine models. These include ovarian [118], bladder [119] and colorectal [120] cancer. The IL-6 axis has been shown to promote mesothelial adhesion and invasion in peritoneal carcinomatosis. For instance, one study found that ovarian cancer cells increased the production of IL-6, which stimulated the production of VEGF, which in turn caused lymphatic vessels to form in the peritoneum, facilitating the spread of cancer cells [121, 122]. During the formation of peritoneal tumor deposits, IL-6 promotes neutrophil and macrophage recruitment and activation, leading to amplification of the inflammatory response [123, 124]. IL-6 stimulates the expression of plasminogen activator inhibitor-1 (PAI-1) and the inhibition of tissue-type plasminogen activator (t-PA), leading to the excessive deposition of fibrin and further promoting PC formation [125]. Signaling pathways involved, including phosphoinositide-dependent kinase 1 (PDK1), have been shown in ovarian cancer to promote tumor-mesothelial adhesion, invasion, and angiogenesis via α5β1 integrin and JNK/IL-8 signaling [126, 127]. ICAM-1 and VCAM-1 are cell adhesion molecules that play a crucial role in the binding of cancer cells to mesothelial cells [128]. IL-6 has been shown to increase the expression of ICAM-1 and VCAM-1 on mesothelial cells, thereby promoting adhesion of tumor cells [129]. This process is further facilitated by the presence of TNF-α, another cytokine that is often upregulated in cancer and can synergize with IL-6 to enhance ICAM-1 expression [130].

IL-6 also induces epithelial to mesenchymal transition (EMT), which is a critical step in the development of metastasis [131–134]. During PC formation, the production of inflammatory mediators regulates the extracellular matrix (ECM), and IL-6 is involved in this process by inducing the expression of matrix metalloproteinases (MMPs). IL-6 has been shown to induce the expression of stromal cell-derived factor-1 (SDF-1), a chemokine that is known to promote the recruitment of mesenchymal stem cells (MSCs) and the formation of a premetastatic niche [135]. IL-6 signaling may also drive the proliferation and migration of mesothelial cells and MSCs, thereby contributing to stromal invasion and proliferation by activating the AKT/mTOR pathway and increasing the expression of cyclin D1 [136–138]. IL-6 potentiates angiogenesis in PC by increasing the expression of VEGF on mesothelial cells, along with stimulating proliferation and migration of endothelial cells, which may contribute to angiogenesis in the tumor microenvironment [139].

The immune response plays a crucial role in the development and progression of PC. As a pivotal mediator of innate immunity, IL-6 suppresses adaptive immune responses within the peritoneum, creating a maladaptive immune environment that enables evasion by tumor cells in peritoneal cancer and mesothelioma. In these diseases, higher IL-6 levels are correlated with more advanced disease stage, increased tumor aggressiveness, and worse clinical outcomes [140]. IL-6 has been shown to inhibit the differentiation of T cells into Th1 cells, which are central to specific cytotoxic anti-tumor immunity; and to promote the differentiation of CD4 + T cells into Th2 and Th17 cells, which are less effective at controlling tumor growth. IL-6 has also been shown to promote the differentiation of regulatory T cells, which suppress the immune response and promote tumor growth [141–143], although the relevance of this finding in the peritoneal cavity remains to be assessed. The cytokine milieu in the tumor microenvironment tends toward promotion of immunosuppressive tumor-associated macrophages (TAMs) [144]. Local cytokines such as CSF-1 block the maturation of dendritic cells, which are unable to present antigens and therefore promote the development of immunosuppressed TAMs. Inhibition of IL-6 and CSF-1 can reverse this effect and favor cytotoxic T cell polarization [145]. Experimental administration of IL-6 inhibitors or receptor antagonists has been shown to reduce tumor growth increase the activity of cytotoxic T cells or natural killer cells in mouse models of PC [146–148]. As a pleiotropic factor, IL-6 may play both pro-tumorigenic and anti-tumorigenic effects, however, and in other studies has been shown to promote the activity of natural killer cells and enhance the immune response to PC [149, 150].

Although not specific to PC, cachexia commonly accompanies end-stage peritoneal progression, and is characterized by the breakdown of carbon sources, proteins, and lipids for energy due to hypercatabolism. This systemic manifestation of cancer is attributed to circulaing cytokines [151, 152], IL-6 being central among them. IL-6 inhibits lipid biosynthesis and promote muscle atrophy and increased catabolism. However, research using IL-6 transgenic mice has been equivocal, and IL-6-induced cachexia appears to require additional signals in what is presumed to be a highly complex process [153–155].

**Fig. 2:**
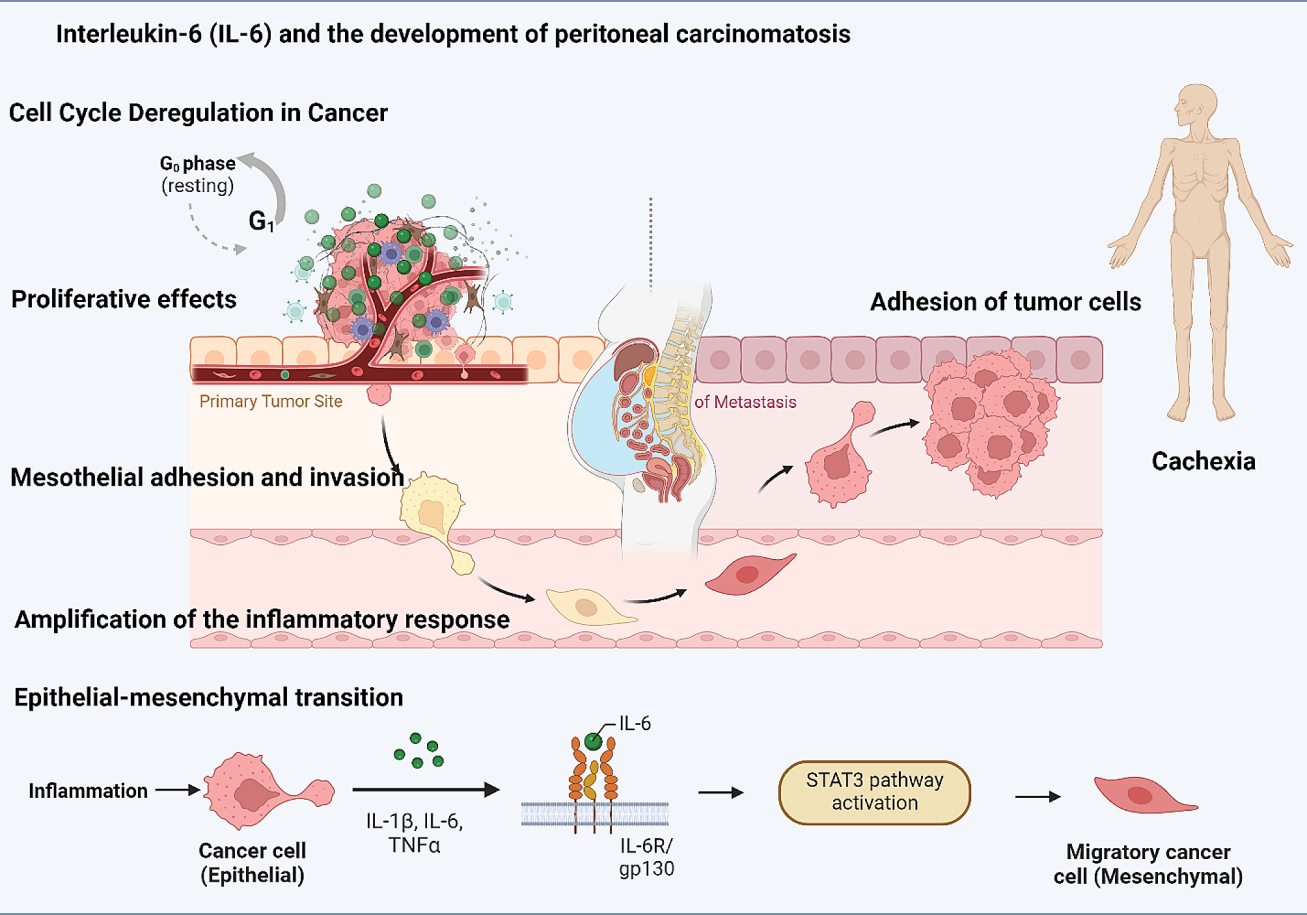
Illustrated here is the crucial role of IL-6 in driving peritoneal carcinomatosis (PC) progression. It shows IL-6’s involvement in tumor proliferation, adhesion, epithelial-mesenchymal transition (EMT), and other oncogenic pathways. The figure spotlights the IL-6/STAT3 signaling pathway’s redundancy and its systemic effects, emphasizing molecular alterations that lead to PC’s aggressive nature. “Created with BioRender.com.”

Taken together, these mechanisms make the IL-6 axis an attractive target for the unique characteristics of PC as a specific pattern of metastatic disease and cancer progression. These common effects of IL-6 provide insights into the shared mechanisms underlying the pathogenesis and progression of these diverse peritoneal diseases. Targeting IL-6 and its associated pathways represents a potential therapeutic approach to mitigate inflammation, modulate immune responses, reduce fibrosis, and improve patient outcomes in these conditions fig. 2.

### The use of targeted therapy for interleukin-6 (IL-6) in the treatment of peritoneal carcinomatosis

Therapy for IL-6 has emerged as a promising approach for the prevention and treatment of PC [79, 156, 157]. Tocilizumab (Actemra) and siltuximab (Sylvant) are the two FDA- approved anti-IL6 drugs with tocilizumab approved for the treatment of rheumatoid arthritis(RA) [158], it has been investigated in the context of cancer for its potent anti-inflammatory effects like large-cell lung carcinoma [159] and siltuximab approved for the treatment of multicentric Castleman’s disease [160]. Other agents in clinical trials include sarilumab [161], olokizumab [162], elsilimomab [163], clazakizumab [164], sirukumab [165], levilimab, CPSI-2364, ALX-0061, and ARGX-109, while preclinical agents include FE301 and FM101 [37]. These agents have shown promising results in various cancers, including ovarian cancer, renal cell carcinoma, and metastatic castration-resistant prostate cancer (Table 1). Mechanistically, blockade of trans signaling accounts for the anti-proliferative effect in certain PC cell lines, along with de-repression of tumor suppressor genes, such as maspin, which impedes stromal invasion and mestastasis [166].

Targeted therapy against IL-6 has shown promising results in mouse models and in vitro cell lines of various cancers, by dampening the IL-6/JAK/STAT3 signaling pathway in gastric cancer [167], ovarian cancer [107, 112, 168–170], and pancreatic cancer [171]. Beyond specific anti-neoplastic activities, targeting IL-6 may benefit patients with PC by preventing the formation of post-operative adhesions [172] or ascites [173]. Pre-clinical and clinical studies have also demonstrated benefit of therapy for IL-6 in cancers not associated with PC. Inhibition of IL-6R function by tocilizumab was shown to decrease angiogenesis in oral squamous cell carcinoma [174]. IL-6 induced programmed death ligand 1 expression through the mTOR pathway in intrahepatic cholangiocarcinoma, suggesting that IL-6 antibodies may help to overcome resistance to immune checkpoint inhibitors in intrahepatic cholangiocarcinoma [175]. Tocilizumab has also been shown to inhibit tumor growth in trastuzumab-resistant breast cancer cells and is the subject of ongoing phase I clinical trials [164, 176]. Tocilizumab has been proposed as a potential treatment option of cancer-related cachexia in lung and other cancers [177]. It has also been indicated for steroid-resistant immune-related adverse events [178]. Below, we have summarized pre-clinical studies and clinical trials examining IL-6 pathway blockade in cancers that are commonly associated with PC.

### Ovarian cancer

Ovarian cancer is one of the leading causes of PC, often in the context of recurrent disease with poor prognosis and limited treatment options. An association between IL-6 and ovarian cancer has been described in the literature for over 30 years, beginning with the observation of IL-6 production by ovarian cancer cell lines, primary ovarian tumors and malignant ascitic fluid [179]. Treating ovarian cancer cell lines with an antisense IL-6 oligodeoxynucleotide resulted in decreased IL-6 production as well as an approximately 80% inhibition in cellular proliferation [180]. Further studies demonstrated that IL-6 levels were significantly higher in ascitic fluid than in the serum of ovarian cancer patients, and the IL-6 levels correlated with higher ascites volume, tumor burden, and worse overall survival [181–183]. Guo et al. noted an increase in IL-6 expression in recurrent ovarian tumors relative to their matching primary, suggesting a role for IL-6 in disease progression and metastasis [161]. There is also data suggesting that IL-6 is involved in chemoresistance in ovarian cancer, with higher levels of IL-6 seen after treatment with platinum chemotherapy in in vitro and murine models [184]. Finally, emerging data from several groups indicate that IL-6 signalling may be critical to EMT in epithelial ovarian cancer [113, 121, 185]. Based on these findings, IL-6 and its pathway have gained increasing interest as a potential therapeutic target in ovarian cancer.

Translational research and clinical trials of IL-6 pathway inhibition in ovarian cancer have had mixed results, but on the balance provide hope for a potential synergistic role of IL-6 inhibition with standard chemotherapy (Table S1). Ovarian cancer cell lines treated with siltuximab monotherapy did not show reduced cell proliferation or protein expression [122, 161], whereas multiple murine models with intraperitoneal ovarian cancer xenografts treated with siltuximab or tocilizumab monotherapy did show a significant impediment to tumor progression [122, 186, 187]. This could reflect the importance of IL-6 inhibition in the physiologic context of the intraperitoneal tumor environment. IL-6 pathway blockade using siltuximab or tocilizumab enhanced the effect of chemotherapy in multiple in vitro and murine in vivo studies [82, 187, 188]. Guo et al., however, did not demonstrate any notable effect on tumor growth with paclitaxel and siltuximab in xenograft mouse models of paclitaxel-resistant ovarian cancer cells [161]. Additional translational studies have been performed with non-antibody IL-6 inhibitors such as butein [189], a compound with anti-IL-6 activity derived from *Butea monosperma* flowers; bazedoxifene, a third-generation selective estrogen receptor modifier (SERM) found to be a novel inhibitor of the IL-6/GP130 interaction [190]; minocycline, a second-generation tetracycline found to have an inhibitory effect on IL-6 signaling; and sgp130Fc, a protein that inhibits IL-6 trans-signaling [173]. Table S1 highlights the mechanisms by which these agents impaired metastasis in vitro and in vivo.

Four clinical trials have examined the use of monoclonal antibody IL-6 pathway inhibitors in treating ovarian cancer. Coward et al. performed a single-arm, phase II clinical trial with platinum-resistant recurrent ovarian cancer patients, in which 18 patients were treated with siltuximab monotherapy. A 5.6% overall response rate was noted, with eight additioanl patients having stable disease. Median progression free survival was 12 weeks, similar to other alternate line chemotherapy agents [122]. Dijkgraaf et al. conducted a multi-center, phase I clinical trial with carboplatin/doxorubicin and tocilizmab in recurrent epithelial ovarian cancer patients. 23 patients were treated in a dose-escalation fashion and an acceptable safety profile was noted. Levels of IL-6 and sIL-6Rα increased with the higher doses of tocilizmab, and CRP and TNF-α levels decreased; however, no efficacy data were available [191]. Angevin et al. conducted a phase I/II, dose escalation study with siltuximab in patients with advanced solid tumors, including 29 patients with ovarian cancer, but an objective tumor response was not observed in any subgroup [192]. Stone et al. evaluated siltuximab in ovarian cancer patients in the setting of paraneoplastic thrombocytosis. Patients were treated with siltuximab and a significant reduction in platelet count was observed, but tumor response was not evaluated [187]. Overall, these studies have shown little promise for the utility of IL-6 pathway inhibition as a standalone treatment for ovarian cancer, while supporting the possibility that combination regimens may be a fertile area for future investigation.

### Gastric cancer

PC is a common complication of advanced gastric cancer that is associated with poor prognosis and limited treatment options. Targeted therapy against the IL-6 pathway has emerged as a promising approach for the prevention and treatment of PC metastasis from gastric cancer, based largely on pre-clinical studies [193, 194]. Kinoshita et al. explored the role of IL-6 in mediating epithelial-stromal interactions and promoting gastric tumorigenesis via crosstalk between epithelial and stromal cells within the gastric microenvironment, contributing to the development of gastric cancer [195]. In another study, STAT3 signaling was found to enhance mesothelial-mesenchymal transition, particularly within the peritoneal cavity [132]. Pre-operative serum IL-6 and CRP levels are associated with poor prognosis in patients with operable gastric cancer, suggesting potential prognostic utility as biomarkers [196]. Ruzzo et al. found that patients harboring genetic variants resulting in up-regulation of IL-6 pathway levels experienced poorer overall survival in GC [197]. Further, elevated perioperative IL-6 and TNF-α levels are negatively associated with 5-year survival in patients with locally advanced GC [198].

As in ovarian cancer, there are theoretical benefits to combining anti-IL-6 therapy with chemotherapy for GC-PC. For example, Wang et al. investigated the tumor-suppressive effects of maslinic acid on human gastric cells, demonstrating that maslinic acid potentiated apoptosis of GC cells in a JAK/STAT3-pathway-dependent mechanism [199]. However, at the present time no clinical trials have reported the use of IL-6 inhibition in the treatment of GC (Table S2).

### Colorectal cancer

IL-6 pathway inhibition has been explored in treating PC from colorectal cancer (CRC) [157], largely as a result of the repeated demonstration that the downstream JAK/STAT3 signaling plays a central role in CRC progression by causing downstream overexpression of VEGF-A and matrix metalloproteinase A (MMP2) [157, 200]. IL-6 trans-signaling has been shown to drive cellular proliferation and inhibit apoptosis in murine models of CRC [201–203]. CRC-derived mesenchymal stem cells were shown to enhance CRC cell migration, invasion through EMT, and metastasis; each of these phenotypes was abrogated by anti-IL6 antibody and STAT3 inhibitors, and was associated with downstream PI3K/AKT signaling [204]. The source of IL-6 within the CRC microenvironment may include tumor cells themselves [35], as well as TAMs [205] and cancer-associated fibroblasts (CAFs) [206]. Li et al. demonstrated a positive feedback loop promoting IL-6 production by macrophages and CRC cells, via a STAT3-dependent mechanism [207]. Yin et al. highlighted the role of macrophage-derived IL-6 in chemoresistance in CRC, showing that miR-155-5p/C/EBPβ/IL6 signaling in TAMs induced chemoresistance via the IL6R/STAT3/miR-204-5p axis in CRC cells [208]. CAF-derived IL-6 was shown to promote angiogenesis by upregulating VEGFA expression in two independent studies, implying a rationale for combining IL-6 inhibition with angiogenesis inhibitors in treating CRC [93, 209].

IL-6 inhibition has been investigated in a number of pre-clinical CRC models (Table S3). Anti-IL-6 antibody administration hindered CRC progression by down-modulating the Ras/MAPK and PI3K/AKT signaling in a murine model [210]. In another murine model of colitis-associated CRC, anti-IL-6 antibody treatment significantly inhibited tumor growth and was associated with downregulation of the pleiotropic transcription factor HIF-1α [211]. Jiang et al. explored the effect of luteolin on CRC cells, confirming suppression of growth and migration/invasion by inhibiting the IL-6/STAT3 signaling pathway [212]. Recently, a novel IL-6-targeted antibody-drug conjugate was shown to effectively inhibit the growth of CRC cells in vitro and in vivo [207]. As in other cancer types, combinatorial activity of IL-6 pathway inhibition with cytotoxic chemotherapy has also been explored. Li et al. demonstrated enhancement of 5-FU response in CRC by simultaneously targeting the IL-6/GP130 signaling pathway [207]. Bazedoxifene, a third-generation SERM with IL-6/GP130 inhibitory effects, markedly potentiated the anti-tumor 5-FU activity in vitro and in vivo, implying a potential role for IL-6 pathway inhibition in reversing chemoresistance [207]. Ying et al., in a study focused on CRC stem-like cells, demonstrated that IL-6 or Notch 3 inhibition may be superior to STAT3 inhibition for cancer stem cell-targeting therapies concomitant with anticancer drugs [213].

In spite of a strong rationale, the clinical literature on IL-6 inhibition in CRC is sparse. A clinical trial examining siltuximab monotherapy in solid tumors included 35 CRCs. Among those treated, only three (< 10%) CRC patients experienced stable disease for > 6 weeks, and there were no objective responses. The overall results provided little rationale for continued investigation of IL-6 monotherapy in solid tumors [192].

### Pancreatic cancer

In pancreatic ductal adenocarcinoma (PDAC), another intra-abdominal cancer with a strong propensity toward PC, IL-6 appears to heavily influence pathogenesis and prognosis (Table S4), as has been expertly reviewed by van Duijneveldt et al. [214]. Elevated serum IL-6 levels are a negative prognostic marker in patients with PDAC, and are consistently associated with advanced clinical stage and decline in nutritional and functional status [215], suggesting that IL-6 levels could augment traditional markers such as CRP, CEA, and CA19-9 [216]. In the tumor microenvironment, IL-6 is overexpressed in PDAC tumors compared to adjacent normal tissue [217]. This overexpression, originating from various cell types such as cancer cells, PSCs, and TAMs, correlates with reduced survival [218]. Mechanistically, IL-6 exerts its effects on tumor initiation, progression, angiogenesis, immune modulation, and metastasis primarily via the STAT3 pathway [219]. IL-6 stimulates production of angiogenic factors like VEGF, which drives the EMT process and enhances the invasive and metastatic capabilities of PDAC cells [220]. Finally, IL-6 may contribute to a maladaptive immune environment in PDAC through complex mechanisms including stimulation of type 2 cytokine secretion, suppression of dendritic cell differentiation and antigen presentation, and recruitment of TAMs and MDSCs [210, 220–223].

Preclinical and animal model studies have been pivotal in understanding IL-6’s role in PDAC and assessing the effectiveness of anti-IL-6 treatments. Murine models show that IL-6 inhibition enhances the sensitivity of PDAC cells to gemcitabine [217, 224, 225], leading to reduced cell proliferation and invasion, and increased apoptosis. This finding is supported in PDAC cell line xenograft models, where IL-6 knockdown, combined with gemcitabine, significantly reduces tumor burden [224]. Advanced models, such as KrasG12D/+/p53fl/+ mutant mice, demonstrate that IL-6-neutralizing antibodies significantly reduce the growth of early-stage PDAC lesions, highlighting the potential importance of IL-6 in tumor initiation [226]. Combination therapies, including anti-IL-6R and anti-PD-L1 immunotherapy, also showed promising results in reducing tumor growth and improving survival [227]. Inhibition of the IL-6 trans signaling pathway, specifically, may be more effective, as evidenced by more pronounced reduction in PanIN lesions in sgp130Tg mice [224].

Several clinical trials have focused on targeting IL-6 family cytokines and their downstream mediators like JAK1/2 and STAT3 in PDAC treatment [228–230]. Notably, a trial involving the JAK1/2 inhibitor ruxolitinib, combined with capecitabine for patients with metastatic pancreatic cancer post-gemcitabine treatment, showed a marginal increase in median overall survival [231]. Other trials, such as those testing momelotinib [228] and the STAT3 inhibitor napabucasin [232] in combination with standard chemotherapy, did not demonstrate significant clinical benefits or are still ongoing. Trials involving IL-6 inhibition, such as those using siltuximab in combination with PD-L1 inhibition (NCT04191421 [233]), and tocilizumab [234], are currently underway. These trials are expected to provide insights into the potential for inhibiting IL-6 in the treatment of this aggressive malignancy.

#### Malignant peritoneal mesothelioma

Although there has been only recent interest in IL-6 pathobiology in malignant peritoneal mesothelioma (MPM), IL-6 has long been suspected to contribute to pathophysiology of pleural malignant mesothelioma (MM) [235, 236]. IL-6 was initially implicated as a key driver of paraneoplastic symptoms in MM, and was found in higher concentrations in MM effusions relative to malignant effusions from lung adenocarcinoma [237]. However in our series, IL-6 was elevated in both MM and lung cancer pleural effusions, and the IL-6 levels were not statistically different between the two diseases [40].

Early pre-clinical models explored the contribution of IL-6 in pleural effusions to constitutional MM symptoms (Table S5), and cell line experiments suggested that anti-IFN-γ therapy could alleviate these symptoms [238]. Anti-IL-6 pathway therapy was utilized as early as 1995 to treat or reverse constitutional toxicity in murine models, without an effect on tumor growth per se [239]. MM cell lines were later shown to account for at least some of the IL-6 found in pleural fluid, with autocrine trans signaling accounting for cellular proliferation, VEGF production, constitutional symptoms, and subject to differential suppression with cytotoxic agent therapy [240, 241]. An engineered viral gene delivery vector encoding a tocilizumab-based IL-6 receptor inhibitor was shown to reduce VEGF production in MM cells [242].

More recent research has cemented the concept of the IL-6 trans signaling axis as a central driver of a maladaptive immunosuppressive pleural environment in MM, and a potentially ideal target for rational therapy [40, 243]. Analogous to pleural fluid in MM [40], elevated IL-6 levels have been confirmed in peritoneal fluid from patients with MPM, and these levels were shown to correlate with disease volume (peritoneal carcinomatosis index) [244]. Given the exorbitant concentration of IL-6 in malignant ascites from MPM, Judge et al. proposed a model in which tumor and stromal cells within the peritoneal cavity undergo reciprocal, synergistic activation [245]. To our knowledge, no clinical trials to date have investigated the specific utility of anti-IL-6 therapy in MM or MPM.

#### Appendiceal cancer

Mucinous appendiceal neoplasms, while rare, are an important clinical subset of patients with PC [246]. In many cases, patients with low grade histology can experience long-term survival following surgical cytoreduction and intra-peritoneal chemotherapy, even with a significant degree of disease burden at the onset of treatment. Given the overall favorable prognosis of these patients, relative to PC from other gastrointestinal primary tumor sites, it is imperative to focus on novel treatment modalities to provide long-term symptom and disease control for appendiceal carcinomatosis (AC).

At present, little is known of the influence of the IL-6 axis in AC (Table S6). Serum IL-6 levels in a series of 12 AC cases were markedly elevated relative to control individuals with uncomplicated appendicitis. A separate series of gastrointestinal neuroendocrine tumors, including 12 appendiceal tumors, showed frequent expression of IL-6 in these tumors, and a trend toward correlation of IL-6 production with tumor grade [247]. Lohani et al. reported an analysis of peritoneal fluid cytokine levels in 23 patients with AC, and peritoneal fluid IL-6 concentrations in this study were elevated ∼ 200-fold above serum levels, with immunohistochemical staining for IL-6 localized to the tumor stroma [248]. At this time, much pre-clinical and clinical investigative work remains to determine whether IL-6 is a key driver of tumor progression and potential target for directed intervention in patients with AC.

### Common themes and future directions

Despite the promising results of preclinical and clinical studies, the use of anti-IL-6 pathway therapy in the treatment of PC faces several challenges, one of which is the heterogeneity of primary cancer types commonly associated with PC. Moreover, the IL-6 pathway is well-known to have complex and diverse functions in the tumor microenvironment, which may have both pro- and anti-tumorigenic effects [249], underscoring the importance to carefully define molecular predictors of response and the selection of appropriate therapeutic candidates. For example, elevation in peritoneal fluid IL-6 levels could be utilized as a selection criterion for clinical trials targeting the IL-6 pathway. Another challenge that must be addressed is the potential immunosuppressive effect of IL-6-targeted therapy and attendant increased risk of infection [250, 251]. Systemic toxicity of IL-6 blockade could theoretically be mitigated by using intra-peritoneal drug administration. Such an approach would leverage significant pharmacokinetic advantages by allowing supra-physiologic dosing of IL-6 antagonists within the sequestered peritoneal environment. To determine the feasibility of this approach, we recently opened a clinical trial examining the safety and pharmacokinetic profile of intra-cavitary (peritoneal and pleural) delivery of tocilizumab for patients with malignant pleural or peritoneal effusions/ascites (NCT06016179).

The available clinical data across cancer types suggests that IL-6 inhibition as monotherapy is unlikely to provide meaningful oncologic benefit. Nevertheless, a combinatorial benefit with conventional chemotherapy has been strongly suggested in a number of models, and therefore it is hoped that IL-6 pathway inhibition could show benefit in combination regimens either in the de novo setting, or in the context of overcoming chemoresistance at the time of progression through standard-of-care options. It is also speculated that IL-6 pathway inhibition could modulate response to other immunotherapeutic regimens currently in standard or investigational use. As a central cytokine with pleiotropic effects, the IL-6 pathway could be manipulated to potentiate the impact of checkpoint inhibition, local/intra-tumoral injection therapies, or other emerging immunotherapeutic options. Finally, in the context of surgery for PC, the IL-6 pathway is likely to be a central mediator of the inflammatory response to surgery or surgical complications, and to contribute to long term morbidity from adhesive disease and fibrosis that challenges our ability to offer operative approaches in many patients. IL-6 inhibition has been considered as a therapeutic approach to peritoneal dialysis-related fibrosis and endometriosis, for example, and might yield benefit in palliating the debilitating digestive, nutritional and cachectic consequences of PC beyond any direct anti-neoplastic activity.

## Conclusion

Peritoneal carcinomatosis is a devastating sequela of many primary tumor types, with poor prognosis and limited treatment options. A significant body of evidence implicates the IL-6 pathway as a rational therapeutic target for treatment of PC, due to IL-6 involvement in peritoneal tumor dissemination, proliferation, mesothelial adhesion and invasion, stromal invasion, and immune response modulation. Targeting IL-6 and its downstream signaling pathways in combination regimens has shown promise in preclinical and clinical studies for various types of cancer that commonly cause PC. Ongoing pre-clinical and clinical studies are planned to explore the utility of regional IL-6 blockade in patients with PC.

### Electronic supplementary material

Below is the link to the electronic supplementary material.

Supplementary Material 1

Supplementary Material 2

Supplementary Material 3

## Data Availability

Not applicable.

## Abbreviations

AC: Appendiceal Cancer
BZA: Bazedoxifene
CAFs: Cancer-Associated Fibroblasts
CRC: Colorectal Cancer
CRP: C-Reactive Protein
EMT: Epithelial-Mesenchymal Transition
GC: Gastric Cancer
IL-1β: Interleukin 1 Beta
IL-6: Interleukin-6
IL-6R: Interleukin-6 Receptor
JAK-STAT: Janus Kinase-Signal Transducer and Activator of Transcription
LN: Lymph Node
MAPK: Mitogen-Activated Protein Kinase
MDSCs: Myeloid-Derived Suppressor Cells
mIL-6Rα: Membrane-Bound Interleukin-6 Receptor Alpha
MM: Malignant Mesothelioma
MMPs: Matrix Metalloproteinases
MPM: Malignant Peritoneal Mesothelioma
MSCs: Mesenchymal Stem Cells
NF-κB: Nuclear Factor Kappa-light-chain-enhancer of activated B cells
OS: Overall Survival
PDAC: Pancreatic Ductal Adenocarcinoma
PI3K-Akt: Phosphoinositide 3-Kinase-Protein Kinase B
PSCs: Pancreatic Stellate Cells
sIL-6Rα: Soluble IL-6 Receptor Alpha
SERM: Selective Estrogen Receptor Modifier
STAT3: Signal Transducer and Activator of Transcription 3
TAMs: Tumor-Associated Macrophages
TNF-α: Tumor Necrosis Factor Alpha
VEGF: Vascular Endothelial Growth Factor
5-FU: 5-Fluorouracil
