# Supplementary material for: Targeting interleukin-6 as a treatment approach for peritoneal carcinomatosis
Source: J Transl Med. 2024 Apr 30;22:402. doi: 10.1186/s12967-024-05205-8 (PMC11061933; doi:10.1186/s12967-024-05205-8)
Supplement: Supplementary file 3 — Supplementary Material 3 [file 12967_2024_5205_MOESM3_ESM.docx]

**Title: Targeting Interleukin-6 as a Treatment Approach for Peritoneal Carcinomatosis**

Table S1-The Role of IL-6 in Ovarian Cancer - Insights from Pre-clinical, Animal Model, and Clinical Studies

| Ovarian | | | | |
| --- | --- | --- | --- | --- |
| Pre-clinical | | | | |
| Author | Title | Date | Summary | Ref. |
| Watson JM, et al. | Constitutive production of interleukin 6 by ovarian cancer cell lines and by primary ovarian tumor cultures | 1990 | Production of IL-6 noted in multiple ovarian cell lines as well as in primary ovarian tumors. | ([1](#_ENREF_1)) |
| Watson JM, et al. | Growth inhibition of ovarian cancer cells induced by antisense IL-6 oligonucleotides | 1992 | Treated ovarian cancer cell lines with an antisense IL-6 oligodeoxynucleotide, which resulted in decreased IL-6 production and inhibition of cellular proliferation. | ([2](#_ENREF_2)) |
| Plante M, et al. | Interleukin-6 level in serum and ascites as a prognostic factor in patients with epithelial ovarian cancer | 1994 | Serum and ascitic fluid specimens collected from patients with newly diagnosed epithelial ovarian cancer. IL-6 levels were significantly higher in ascitic fluid than in serum. The level of IL-6 in ascites was correlated to ascites volume and tumor burden. | ([3](#_ENREF_3)) |
| Scambia G, et al. | Prognostic significance of interleukin 6 serum levels in patients with ovarian cancer | 1995 | Serum collected from 114 ovarian cancer patients. IL-6 level was not significantly related to stage, histology, grade, or presence of ascites. A shorter survival was noted in patients that had higher IL-6 levels. | ([4](#_ENREF_4)) |
| Guo Y, et al. | Effects of siltuximab on the IL-6-induced signaling pathway in ovarian cancer | 2010 | Ovarian cancer cell lines were treated with siltuximab and a decrease in Stat3 phosphorylation and nucleocytoplasmic translocation was noted, which lead to reduced levels of BcL-X_L,_ *MCL-1,* and survivin (downstream Stat3 antiapoptotic proteins.) Paclitaxel resistant cell lines treated with siltuximab+paclitaxel were noted to have increased paclitaxel-induced cell death. | ([5](#_ENREF_5)) |
| Coward J, et al. | Interleukin-6 as a therapeutic target in human ovarian cancer | 2011 | Ovarian cancer cell lines (2 lines that produced IL-6 and 2 lines that did not) were treated with siltuximab. There was a reduction in Stat3 phosphorylation as well as the release of cytokines IL-1β, TNF-α, IL-8, and CCL2 in the cell lines that constitutively produced IL-6. There was no change in malignant cell growth in any cell lines. | ([6](#_ENREF_6)) |
| Anglesio MS, et al. | IL6-STAT3-HIF signaling and therapeutic response to the angiogenesis inhibitor Sunitinib in ovarian clear cell cancer | 2011 | Ovarian cancer tissue samples evaluated and noted higher levels of IL-6 and overexpression of the IL6-STAT3-HIF pathway in ovarian clear cell cancer compared to high grade serous ovarian cancer. There was also an association of increased IL-6 and worse progression free survival and overall survival. | ([7](#_ENREF_7)) |
| Lo CW, et al. | IL-6 trans-signaling in formation and progression of malignant ascites in ovarian cancer | 2011 | Malignant ovarian ascites and ovarian cancer cell lines were noted to have significantly increased IL-6Rα expression, the receptor that binds to IL-6 before binding to gp130 and initiating the signaling pathway. | ([8](#_ENREF_8)) |
| Wang Y, et al. | Interleukin-6 signaling regulates anchorage-independent growth, proliferation, adhesion, and invasion in human ovarian cancer cells | 2012 | Ovarian cancer cell lines were studied by overexpressing IL-6 in non-IL-6-expressing ovarian cell lines and depleting IL-6 expression in IL-6-overexpressing cell lines. This demonstrated that IL-6 is involved in the growth, proliferation, adhesion, and invasion of ovarian cancer cells. IL-6 expression also correlated to increased expression of IL-6Rα, gp130, and the phosphorylation of ERK and Akt pathways. | ([9](#_ENREF_9)) |
| Cohen S, et al. | Platinum-resistance in ovarian cancer cells is mediated by IL-6 secretion via the increased expression of its target cIAP-2 | 2013 | Treatment of ovarian cancer cell lines with cisplatin demonstrated a significant increase in IL-6 mRNA, IL-6 protein secretion, and cIAP-2 (an inhibitor of apoptosis protein.) Utilizing an anti-IL-6 antibody was noted to sensitize platinum-resistant ovarian cancer cells to cisplatin. A reduction in cIAP-2 levels was also appreciated. There was no effect on cell viability with the anti-IL-6 antibody alone. | ([10](#_ENREF_10)) |
| Ataie-Kachoie P, et al. | Minocycline suppresses interleukine-6, its receptor system and signaling pathways and impairs migration, invasion, and adhesion capacity of ovarian cancer cells: in vitro and in vivo studies | 2013 | Ovarian cancer cell lines were treated with minocycline (second-generation tetracycline with inhibitory effect on IL-6). Minocycline decreased IL-6 expression in all cell lines. The SKOV-3 cell line was further evaluated and minocycline was found to decrease the expression of IL-6Rα and gp130, the phosphorylation of STAT3, the translocation of STAT3 to the nucleus, as well as expression of the downstream apoptosis inhibitory protein, MCL-1. Minocycline was also noted to decrease expression of MMP-2 and MMP-9 in the SKOV-3 cell line which inhibited the migration and invasion ability of the cells. | ([11](#_ENREF_11)) |
| Isobe A, et al. | Interleukin 6 receptor is an independent prognostic factor and a potential therapeutic target of ovarian cancer | 2015 | Evaluated 94 ovarian cancer tumors and 34% had “high” IL-6 receptor expression which was noted to be associated with worse progression free survival. IL-6 expression was also evaluated and 41.4% showed a “high” IL-6 expression, however this did not have prognostic value. Ovarian cancer cell line was treated with exogenous IL-6 which induced cell invasion, proliferation, and VEGF production. Treatment with an anti-IL-6R antibody decreased this enhancement. | ([12](#_ENREF_12)) |
| Kim S, et al. | Malignant ascites enhances migratory and invasive properties of ovarian cancer cells with membrane bound IL-6 in vitro | 2016 | Ascitic fluid samples of ovarian cancer patients were found to have high levels of IL-6. The SKOV-3 ovarian cancer cell line was treated with the malignant ascitic fluid and an increase in JAK2 and STAT3 phosphorylation was noted, along with increased cell migration and invasion. Treating the ascitic fluid with an IL-6 antibody first suppressed this enhancement. Additional cell lines were evaluated and there was no increase in invasion appreciated with ascitic fluid in 2/3 additional cell lines. | ([13](#_ENREF_13)) |
| Pasquier J, et al. | CCL2/CCL5 secreted by the stroma induce IL-6/PYK2 dependent chemoresistance in ovarian cancer | 2018 | Ovarian cancer cells were stimulated with IL-6 prior to chemotherapy and chemoresistance was noted. Cell lines were then treated with an IL-6 antibody, which was not toxic to the cells and prevented chemoresistance. Mesenchymal stem cells were noted to increase IL-6 secretion by ovarian cancer cells by way of CCL2 and CCL5 cytokine secretion. The study also suggested that PYK2 phosphorylation (a focal adhesion kinase) by IL-6 autostimulation leads to chemoresistance. The PYK2 phosphorylation was increased in the presence of mesenchymal stem cells. | ([14](#_ENREF_14)) |
| Wang Y, et al. | IL-6 mediates platinum-induced enrichment of ovarian cancer stem cells | 2018 | Ovarian cell lines were studied and there was a higher expression of IL-6/IL-6R in ALDH+ cells. STAT3 phosphorylation was associated with expression of ALDH1A1. This can contribute to the enrichment of ovarian cancer stem cells after platinum therapy. Cell lines were treated with guadacitabine and IL-6-Nab alone and together. There was a more effective decrease in ALDH+ ovarian cancer stem cell population with combined therapy. | ([15](#_ENREF_15)) |
| Yousefi H, et al. | IL-6/IL-6R pathway is a therapeutic target in chemoresistant ovarian cancer | 2019 | Multiple ovarian cancer cell lines were treated with tocilizumab+carboplatin vs carboplatin alone. Synergistic effect noted in some cell lines (but not all) with increased antiproliferation in combination therapy. The study findings also suggest that EGFR/ERK/NF-κB and EGFR/PI3K/NF-κB signaling crosstalks induce IL-6 expression in chemoresistant cells. | ([16](#_ENREF_16)) |
| Mehner C, et al. | Targeting an autocrine IL-6 – SPINK1 signaling axis to suppress metastatic spread in ovarian clear cell carcinoma | 2020 | SPINK1 expression in ovarian cancer cells was found to be decreased with IL-6 silencing, demonstrating a regulatory effect by IL-6. Ovarian cancer cell lines were treated with tocilizumab and there was a decrease in STAT3 phosphorylation and SPINK1 expression. This led to decreased cell survival in ultra-low attachment plates. | ([17](#_ENREF_17)) |
| Ge J, et al. | Long non-coding RNA THOR promotes ovarian cancer cells progression via IL-6/STAT3 pathway | 2020 | Long non-coding RNA THOR was shown to be upregulated in ovarian cancer tumor tissue and associated with higher risk of recurrence and shorter survival. THOR knockdown led to inhibited STAT3 phosphorylation and suppressed proliferation of ovarian cancer cell lines. This suggests that THOR expression promotes ovarian cancer cell progression via activating the IL-6/STAT-3 signaling pathway. | ([18](#_ENREF_18)) |
| Zhang R, et al. | Combined inhibition of IL-6 and IL-8 pathways suppresses ovarian cancer cell viability and migration and tumor growth | 2022 | Ovarian cancer cell lines were treated with Bazedoxifene (SERM that inhibits IL-6), SCH527123 (IL-8 inhibitor), and combined. Inhibition in cell proliferation, viability, and migration was noted with each drug. Downstream targeted genes in the IL-6 and IL-8 pathways were also decreased including p-STAT3/STAT3, p-AKT/AKT, p-S6/S6, and surviving/GAPDH. The greatest inhibition was noted with combination therapy. | ([19](#_ENREF_19)) |
| Park S, et al. | Butein inhibits cell growth by blocking the IL-6/IL6Rα interaction in human ovarian cancer and by regulation of the IL-6/STAT3/FoxO3a pathway | 2023 | Butein is isolated from butea monosperma flowers and found to have anti-IL-6 activity. The inhibitory effect is due to a higher binding affinity to IL-6, as well as binding abilities to IL-6Rα and gp130. Butein was found to inhibit cell growth in ovarian cancer cell lines more than treatment with siltuximab. | ([20](#_ENREF_20)) |
| Animal Model | | | |  |
| Guo Y, et al. | Effects of siltuximab on the IL-6-induced signaling pathway in ovarian cancer | 2010 | Paclitaxel-resistant ovarian cancer intraperitoneal xenograft mouse models were treated with siltuximab+paclitaxel. There was no significant effect on tumor growth noted. | ([5](#_ENREF_5)) |
| Coward J, et al. | Interleukin-6 as a therapeutic target in human ovarian cancer | 2011 | Two different ovarian cancer cell lines were used for intraperitoneal xenograft mouse models. One cell line had IL-6 expression and the other did not. The mice were given siltuximab and there was a reduction in malignant cells in the IL-6 producing tumor. | ([21](#_ENREF_21)) |
| Lo CW, et al. | IL-6 trans-signaling in formation and progression of malignant ascites in ovarian cancer | 2011 | Ovarian cancer xenograft mouse models were treated with control, sgp130Fc (selective inhibitor of IL-6 trans-signaling) alone, or sgp130Fc in combination with Taxol. The greatest tumor response was noted in the combination therapy and this therapy completely inhibited measurable ascites. P-STAT3 expression was analyzed from tumor biopsies and confirmed decreased expression with sgp130Fc treatment. | ([8](#_ENREF_8)) |
| Stone R, et al. | Paraneoplastic thrombocytosis in ovarian cancer | 2012 | Ovarian cancer xenograft mouse models were treated with siltuximab, paclitaxel, or both. The monotherapies had significant reduction in tumor growth, but the combination therapy was the most effective. | ([22](#_ENREF_22)) |
| Ataie-Kachoie P, et al. | Minocycline suppresses interleukine-6, its receptor system and signaling pathways and impairs migration, invasion, and adhesion capacity of ovarian cancer cells: in vitro and in vivo studies | 2013 | Xenograft ovarian cancer mouse models were given minocycline and a considerable decrease in plasma and tumor levels of IL-6 was demonstrated. The tumor expression of phosphorylated STAT3 and MCL-1 was also inhibited. | ([11](#_ENREF_11)) |
| Pasquier J, et al. | CCL2/CCL5 secreted by the stroma induce IL-6/PYK2 dependent chemoresistance in ovarian cancer | 2018 | Ovarian cancer xenograft mouse models were utilized and some of the models were given mesenchymal stem cells (MSC). They were then all treated with chemotherapy +/- tocilizumab. Mice injected with MSC had notable chemoresistance. Combination treatment with tocilizumab had a greater response rate than chemotherapy alone. | ([14](#_ENREF_14)) |
| Wang Y, et al. | IL-6 mediates platinum-induced enrichment of ovarian cancer stem cells | 2018 | Intraperitoneal xenograft mouse model of platinum-sensitive ovarian cancer cells was treated with carboplatin. Increased IL-6 levels and IL-6R expression was noted. Combination treatment of guadecitabine+IL-6-Nab was more effective in treating tumor relapse after prior platinum therapy. | ([15](#_ENREF_15)) |
| Mehner C, et al. | Targeting an autocrine IL-6 – SPINK1 signaling axis to suppress metastatic spread in ovarian clear cell carcinoma | 2020 | SPINK1 was knocked down in ovarian cancer xenograft mouse models and tumor growth was decreased compared to the control mice. Mouse models were then treated with tocilizumab. There was a significant reduction in ascites volume and tumor metastasis in the diaphragm and omentum compared to the control mice. There was a trend toward reduced SPINK1 in the tocilizumab treated mice. | ([17](#_ENREF_17)) |
| Ge J, et al. | Long non-coding RNA THOR promotes ovarian cancer cells progression via IL-6/STAT3 pathway | 2020 | THOR knockdown was performed on ovarian cancer xenograft mouse models and was found to inhibit ovarian cancer cell growth. | ([18](#_ENREF_18)) |
| Zhang R, et al. | Combined inhibition of IL-6 and IL-8 pathways suppresses ovarian cancer cell viability and migration and tumor growth | 2022 | Ovarian cancer xenograft mouse models were treated with Bazedoxifene, SCH527123, or combination. Significant reduction in tumor growth was noted with each drug and even more reduction with combination therapy. | ([23](#_ENREF_23)) |
| Park S, et al. | Butein inhibits cell growth by blocking the IL-6/IL6Rα interaction in human ovarian cancer and by regulation of the IL-6/STAT3/FoxO3a pathway | 2023 | Ovarian cancer xenograft mouse models were treated with butein. Butein demonstrated significant inhibition of tumor growth compared to placebo or siltuximab. | ([20](#_ENREF_20)) |
| Clinical Trial | | | |  |
| Coward J, et al. | Interleukin-6 as a therapeutic target in human ovarian cancer | 2011 | Single-arm, phase II clinical trial of platinum-resistant recurrent ovarian cancer and siltuximab treatment. 18 patients treated: 8 with progression on treatment, 10 with stable disease (one of which with possible partial-response). | ([21](#_ENREF_21)) |
| Stone R, et al. | Paraneoplastic thrombocytosis in ovarian cancer | 2012 | Ovarian cancer patients with paraneoplastic thrombocytosis were treated with siltuximab. Significant reduction in platelet count was noted, but tumor response was not evaluated. | ([22](#_ENREF_22)) |
| Angevin E, et al. | A phase I/II, multiple-dose, dose-escalation study of siltuximab, an anti-interleukin-6 monoclonal antibody, in patients with advanced solid tumors | 2014 | Phase I/II, dose-escalation clinical trial of advanced solid tumors treated with siltuximab. 29/84 patients had ovarian cancer, but no objective tumor response could be evaluated. | ([24](#_ENREF_24)) |
| Dijkgraaf EM, et al. | A phase I trial combining carboplatin/doxorubicin with tocilizumab, an anti-IL-6R monoclonal antibody, and interferon-α2b in patients with recurrent epithelial ovarian cancer | 2015 | Multi-center, phase I, dose-escalation clinical trial of progressive/relapsed epithelial ovarian cancer and carboplatin/doxorubicin with Tocilizumab. Appropriate safety profile was demonstrated, but the trial was not designed to evaluate tumor response. | ([25](#_ENREF_25)) |

Table S2-Insights from Gastric Cancer Research - Pre-clinical, Animal Model, and Clinical Studies

| Gastric | | | | |
| --- | --- | --- | --- | --- |
| Pre-clinical | | | | |
| Author | **Title** | **Date** | **Summary** |  |
| Kim, D., et al. | Clinical significances of preoperative serum interleukin-6 and C-reactive protein level in operable gastric cancer | 2009 | Preoperative serum IL-6 and CRP levels were evaluated as markers of tumor invasion, LN metastasis, and TNM stage. Preoperative high IL-6 levels were proposed as a poor prognostic factor for disease recurrence and overall survival in patients with gastric cancers. | ([26](#_ENREF_26)) |
| Ruzzo, A., et al. | Genetic modulation of the interleukin 6 (IL-6) system in patients with advanced gastric cancer: a background for an alternative target therapy | 2014 | Genetic variants that up-regulate the IL-6 system showed impact on OS (Overall Survival). | ([27](#_ENREF_27)) |
| Wang, D., et al. | Maslinic acid suppresses the growth of human gastric cells by inducing apoptosis via inhibition of the interleukin-6 mediated Janus kinase/signal transducer and activator of transcription 3 signaling pathway | 2017 | Maslinic acid treatment significantly reduced cell proliferation, induced apoptosis and was accompanied by a significant decrease in Bcl-2, Bax and Bad expression levels. Maslinic acid treatment also resulted in the downregulation of phosphorylated-STAT3 and JAK2, and significantly inhibited the protein expression of IL-6. Maslinic acid is able to inhibit MKN28 cell proliferation and the phosphorylation of STAT3 by downregulating the expression of IL-6. These results suggest that maslinic acid suppresses the growth of MKN28 cells by inducing apoptosis via its inhibition of the IL-6/JAK/STAT3 signaling cascade. | ([28](#_ENREF_28)) |
| Ham, I., et al. | Targeting interleukin-6 as a strategy to overcome stroma-induced resistance to chemotherapy in gastric cancer | 2019 | IL-6 is a key contributor to chemoresistance. | ([29](#_ENREF_29)) |
| Zhang, Z., et al. | Effect of Perioperative Interleukin-6 and Tumor Necrosis Factor- α on Long-Term Outcomes in Locally Advanced Gastric Cancer: Results from the CLASS-01 Trial | 2022 | Perioperative high serum IL6 and TNFα levels are negatively associated with 5-year survival outcomes in patients with locally advanced gastric cancer, indicating the potential survival benefits from perioperative anti-inflammatory treatment. | ([30](#_ENREF_30)) |
| Animal Model | | | |  |
| Kinoshita, H., et al. | Interleukin-6 mediates epithelial-stromal interactions and promotes gastric tumorigenesis | 2013 | Importance of IL-6 mediated stromal-epithelial cell interaction in gastric tumorigenesis | ([31](#_ENREF_31)) |
| Yang, H., et al. | STAT3 promotes peritoneal metastasis of gastric cancer by enhancing mesothelial-mesenchymal transition | 2021 | STAT3 activation was found to contribute to peritoneal metastasis of Gastric Cancer by promoting MMT (Mesothelial to Mesenchymal Transition). | ([32](#_ENREF_32)) |

Table S3-Exploring Colorectal Cancer - Insights from Pre-clinical and Animal Model Studies

| COLORECTAL | | | |  |
| --- | --- | --- | --- | --- |
| Pre-clinical | | | |  |
| Author | Title | Date | Summary |  |
| Li, Y., et al. | Interleukin-6 (IL-6) released by macrophages induces IL-6 secretion in the human colon cancer HT-29 cell line | 2009 | Analyzed the surgical specimens of 126 patients with colon cancer and demonstrated the colocalization of macrophages and the expression of IL-6 in colon cancer patients and indicate that macrophages in tumor infiltrates could release IL-6, which in turn conditions colon cancer cells, causing them to secrete IL-6 themselves via phosphorylation of STAT3. | ([33](#_ENREF_33)) |
| Hsu, C., et al. | Anti-interleukin-6 receptor antibody inhibits the progression in human colon carcinoma cells | 2011 | IL-6 and the IL-6R complex could induce clonogenic growth and invasiveness by mediating signals in the Ras/MAPK and PI3K/AKt pathways, and the malignant phenotypes might be associated with the production of MMP-2 and MMP-9 after IL-6 stimulation in SW480 cancer cells. | ([34](#_ENREF_34)) |
| Ying, J., et al. | The effectiveness of an anti-human IL-6 receptor monoclonal antibody combined with chemotherapy to target colon cancer stem-like cells | 2015 | Anti-human IL-6 receptor monoclonal antibody or Notch 3 inhibition may be superior to STAT3 inhibition for Cancer stem cell-targeting therapies concomitant with anticancer drugs. | ([35](#_ENREF_35)) |
| Han, J., et al. | Interleukin-6 promotes tumor progression in colitis-associated colorectal cancer through HIF-1α regulation | 2016 | Anti-IL-6 receptor antibody treatment significantly inhibited the development of colorectal cancer and the expression of HIF-1α, in colorectal adenomas and adenocarcinomas. The study suggests that IL-6 promotes progression through HIF-1α regulation. | ([36](#_ENREF_36)) |
| Yin, Y., et al. | The Immune-microenvironment Confers Chemoresistance of Colorectal Cancer through Macrophage-Derived IL6 | 2017 | Maladjusted miR-155-5p/C/EBPβ/IL6 signaling in tumor-associated macrophages could induce chemoresistance in colorectal cancer cells by regulating the IL6R/STAT3/miR-204-5p axis | ([37](#_ENREF_37)) |
| Zhang, X., et al. | Human colorectal cancer-derived mesenchymal stem cells promote colorectal cancer progression through IL-6/JAK2/STAT3 signaling | 2018 | Human colorectal cancer-derived MSCs (CC-MSCs) increased the migration and invasion of colorectal cancer cells and promoted the tumorigenesis of colorectal cancer through epithelial-to-mesenchymal transition (EMT) in vitro, as well as enhanced the growth and metastasis of colorectal cancer in vivo. It was determined that interleukin-6 (IL-6) was the most highly expressed cytokine in the CC-MSC conditioned medium and promoted the progression of colorectal cancer cells through IL-6/JAK2/STAT3 signaling, which activated PI3K/AKT signaling. | ([38](#_ENREF_38)) |
| Li, J., et al. | Targeting Interleukin-6 (IL-6) Sensitizes Anti-PD-L1 Treatment in a Colorectal Cancer Preclinical Model | 2018 | IL-6 induces strong immunosuppression in the CRC microenvironment by recruiting immunosuppression cells and impairing T cell infiltration. Inhibition of IL-6 enhanced the efficacy of anti-PD-L1 in CRC, providing a novel strategy to overcome anti-PD-L1 resistance in CRC. | ([39](#_ENREF_39)) |
| Li, S., et al. | Down-regulating IL-6/GP130 targets improved the anti-tumor effects of 5-fluorouracil in colon cancer | 2018 | Bazedoxifene (BZA), a third-generation selective estrogen receptor modulator, was discovered to have a novel function as an IL-6/GP130 target inhibitor. Thus, speculated that in colon cancer, the anti-tumor efficacy of 5-FU might be increased in combination with IL-6/GP130 inhibitors. BZA markedly potentiates the anti-tumor function of 5-FU in vitro and in vivo. This study firstly verifies that targeting IL-6/GP130 signaling can increase the anti-tumor function of 5-FU; in addition, this strategy can sensitize cancer cell drug sensitivity, implying that blocking IL-6/GP130 targets can reverse chemoresistance. | ([40](#_ENREF_40)) |
| Elkazzaz, M., et al. | Inhibition of colorectal cancer targets IL-6, CTLA-4, & B7-2 by Tislelizumab: molecular docking, dynamics, & STRING protein-protein network analysis | 2023 | Tislelizumab's molecular docking and dynamics simulations with the receptor B7-2(CD86) and its downstream signaling proteins CTLA-4 and IL-6. | ([41](#_ENREF_41)) |
| Animal model | | | |  |
| Nagasaki, T., et al. | Interleukin-6 released by colon cancer-associated fibroblasts is critical for tumor angiogenesis: anti-interleukin-6 receptor antibody suppressed angiogenesis and inhibited tumor-stroma interaction | 2014 | Cancer stromal fibroblasts were an important source of IL-6 in colon cancer. IL-6 produced by activated fibroblasts induced tumor angiogenesis by stimulating adjacent stromal fibroblasts. | ([42](#_ENREF_42)) |
| Heichler, C., et al. | STAT3 activation through IL-6/IL-11 in cancer-associated fibroblasts promotes colorectal tumor development and correlates with poor prognosis | 2020 | pSTAT3 expression in Cancer-associated fibroblasts (CAFs) of human TMAs revealed a negative correlation of increased stromal pSTAT3 expression with the survival of colon cancer patients. The blockade of proangiogenic signaling significantly reduced colorectal tumor growth in mice with constitutive STAT3 activation in COLVI+ fibroblasts | ([43](#_ENREF_43)) |

Table S4-Exploring Pancreatic Cancer - Insights from Pre-clinical and Animal Model Studies

| Pancreatic Cancer | | | | |
| --- | --- | --- | --- | --- |
| Preclinical | | | | |
| Author | **Title** | **Date** | **Summary** | **Ref** |
| Zhang,H., et al. | Paracrine SDF-1α signaling mediates the effects of PSCs on GEM chemoresistance through an IL-6 autocrine loop in pancreatic cancer cells | 2015 | Study demonstrated that autocrine signaling of IL-6 by pancreatic cancer cells prevents gemcitabine-induced apoptosis. IL-6 knockdown increased sensitivity to gemcitabine, reducing proliferation and invasion. | ([44](#_ENREF_44)) |
| Xing,H, et al. | Suppression of IL-6 Gene by shRNA Augments Gemcitabine Chemosensitization in Pancreatic Adenocarcinoma Cells | 2018 | Investigation of IL-6 knockdown in PDAC cell lines showing reduced tumor burden when combined with gemcitabine. Highlights the therapeutic potential of targeting IL-6 in PDAC. | ([45](#_ENREF_45)) |
| Animal model | | | | |
| Lesina,M., et al. | Stat3/Socs3 Activation by IL-6 Trans signaling Promotes Progression of Pancreatic Intraepithelial Neoplasia and Development of Pancreatic Cancer | 2011 | Analysis of IL-6 signaling pathways in vivo, showing that targeting trans-signaling might be more effective in reducing PanIN lesions in PDAC. | ([46](#_ENREF_46)) |
| Zhang,Y., et al. | Interleukin-6 Is Required for Pancreatic Cancer Progression by Promoting MAPK Signaling Activation and Oxidative Stress Resistance | 2013 | Treatment with an IL-6 neutralizing antibody led to reduced ADM and PanIN lesions in a mouse model of PDAC, suggesting the effectiveness of IL-6 inhibition in early disease stages. | ([47](#_ENREF_47)) |
| Goumas,F., et al. | Inhibition of IL-6 signaling significantly reduces primary tumor growth and recurrencies in orthotopic xenograft models of pancreatic cancer | 2015 | Explored the efficacy of anti-IL-6R and sgp130 treatments in PDAC xenografts, finding reduced tumor burden and metastatic spread. | ([48](#_ENREF_48)) |
| Mace,T., et al. | IL-6 and PD-L1 antibody blockade combination therapy reduces tumor progression in murine models of pancreatic cancer | 2018 | Study found that combining anti-IL-6R with anti-PD-L1 immunotherapy reduced tumor growth and increased survival in PDAC mouse models, indicating a synergistic effect. | ([49](#_ENREF_49)) |
| Rupert,J ., et al. | Tumor-derived IL-6 and trans-signaling among tumor, fat, and muscle mediate pancreatic cancer cachexia | 2021 | This study linked muscle wasting and mortality in PDAC patients to IL-6 levels, using patient-derived xenografts. PDAC cells were found to induce IL-6 in adipocytes and myocytes, causing tissue wasting. Reducing IL-6 in cancer cells significantly lessened cachexia, suggesting IL-6 trans-signaling as a therapeutic target. | ([50](#_ENREF_50)) |
| Ware,M., et al. | Dual IL-6 and CTLA-4 blockade regresses pancreatic tumors in a T cell- and CXCR3-dependent manner | 2023 | Dual blockade of IL-6 and CTLA-4 in mice significantly inhibits pancreatic tumor growth by enhancing T-cell infiltration and dependency on the CXCR3 axis. | ([51](#_ENREF_51)) |
| Clinical | | | |  |
| Angevin E., et al. | A phase I/II, multiple-dose, dose-escalation study of siltuximab, an anti-interleukin-6 monoclonal antibody, in patients with advanced solid tumors | 2014 | This research explores the potential of Siltuximab, an anti-IL-6 monoclonal antibody, as a standalone treatment for pancreatic cancer. The trial is currently ongoing, and the results are pending.( NCT00841191) | ([24](#_ENREF_24)) |
| Hurwitz, H., et al. | Ruxolitinib + capecitabine in advanced/metastatic pancreatic cancer after disease progression/intolerance to first-line therapy: JANUS 1 and 2 randomized phase III studies | 2018 | This study examines the effect of ruxolitinib combined with capecitabine in patients with metastatic pancreatic cancer post-gemcitabine treatment. The trial observed a marginal extension in median overall survival but was terminated early due to lack of efficacy.( NCT02117479), ([NCT02119663](http://clinicaltrials.gov/show/NCT02119663)). | ([52](#_ENREF_52)) |
| Ng, K., et al. | Phase 1 dose-escalation study of momelotinib, a Janus kinase 1/2 inhibitor, combined with gemcitabine and nab-paclitaxel in patients with previously untreated metastatic pancreatic ductal adenocarcinoma | 2019 | This clinical trial evaluates the efficacy of momelotinib in combination with gemcitabine and nab-paclitaxel in untreated metastatic pancreatic cancer patients. The study found no significant clinical benefit over the standard dual treatment. | ([53](#_ENREF_53)) |
| Bekaii-saab T., et al. | Napabucasin plus nab-paclitaxel with gemcitabine versus nab-paclitaxel with gemcitabine in previously untreated metastatic pancreatic adenocarcinoma: an adaptive multicentre, randomized, open-label, phase 3, superiority trial | 2023 | The study investigates the effectiveness of Napabucasin, a STAT3 inhibitor, combined with gemcitabine/nab-paclitaxel in treating metastatic pancreatic cancer. The trial is ongoing, focusing on assessing the efficacy and safety of the treatment.( NCT02993731) | ([54](#_ENREF_54)) |
| Chen I., et al. | Randomized phase 2 study of nab-paclitaxel and gemcitabine with or without tocilizumab as first-line treatment in patients with advanced pancreatic cancer (PACTO). | 2023 | The trial evaluates the safety and efficacy of Tocilizumab, an anti-IL-6R monoclonal antibody, in combination with gemcitabine and nab-paclitaxel for treating unresectable locally advanced or metastatic pancreatic cancer. The study is still in progress.( NTC02767557) | ([55](#_ENREF_55)) |

Table S5-Exploring Malignant Peritoneal Mesothelioma- Insights from Pre-clinical and Animal Model Studies

| Pre-clinical | | | | |
| --- | --- | --- | --- | --- |
| Author | **Title** | **Date** | **Summary** | **Ref** |
| Schmitter D., et al. | Hematopoietic growth factors secreted by seven human pleural mesothelioma cell lines: Interleukin-6 production as a common feature | 1992 | Investigates IL-6's role in the pathophysiology of pleural malignant mesothelioma (MM), suggesting its contribution to paraneoplastic symptoms. | ([56](#_ENREF_56)) |
| Nakano T., et al. | Interleukin 6 and its relationship to clinical parameters in patients with malignant pleural mesothelioma | 1998 | Study showing higher IL-6 concentrations in MM effusions compared to malignant effusions from lung adenocarcinoma. | ([57](#_ENREF_57)) |
| Adachi Y., et al. | Interleukin-6 induces both cell growth and VEGF production in malignant mesotheliomas | 2006 | Shows MM cell lines contribute to IL-6 in pleural fluid, affecting cell proliferation, VEGF production, and constitutional symptoms. | ([58](#_ENREF_58)) |
| Adachi Y., et al. | VEGF targeting in mesotheliomas using an interleukin-6 signal inhibitor based on adenovirus gene delivery | 2010 | developed an adenovirus-based gene delivery system for NRI, a receptor inhibitor of IL-6, which effectively reduced VEGF production in mesothelioma cells, suggesting its potential as a treatment agent for mesothelioma | ([59](#_ENREF_59)) |
| Clinical | | | |  |
| Vlaeminck-Guillem, V., et al. | Intraperitoneal cytokine level in patients with peritoneal surface malignancies. A study of the RENAPE (French Network for Rare Peritoneal Malignancies) | 2013 | Confirms elevated IL-6 levels in peritoneal fluid of MPM patients, correlating with disease volume. | ([60](#_ENREF_60)) |
| Judge S., et al | Malignant peritoneal mesothelioma: characterization of the inflammatory response in the tumor microenvironment | 2016 | Proposes a model of mutual activation between tumor and stromal cells in MPM, driven by high IL-6 levels. | ([61](#_ENREF_61)) |

Table S6-Exploring Appendiceal Cancer- Insights from Pre-clinical and Animal Model Studies

| Clinical | | | | |
| --- | --- | --- | --- | --- |
| Author | **Title** | **Date** | **Summary** | **Ref** |
| Carr N., et al | The histopathological classification, diagnosis and differential diagnosis of mucinous appendiceal neoplasms, appendiceal adenocarcinomas and pseudomyxoma peritonei | 2017 | IL-6 expression observed in gastrointestinal neuroendocrine tumors, including appendiceal tumors, with a potential correlation with tumor grade | ([62](#_ENREF_62)) |
| Mahečić D., et al | Inflammation-related cytokines and their roles in gastroenteropancreatic neuroendocrine neoplasms | 2020 | Peritoneal fluid IL-6 concentrations in AC patients were dramatically higher than serum levels, and IL-6 was localized to the tumor stroma. | ([63](#_ENREF_63)) |

**References:**

1. Watson JM, Sensintaffar JL, Berek JS, Martínez-Maza O. Constitutive production of interleukin 6 by ovarian cancer cell lines and by primary ovarian tumor cultures. Cancer research. 1990;50(21):6959-65.

2. Watson J, Berek J. Growth inhibition of ovarian cancer cells induced by antisense IL-6 oligonucleotide-mediated IL-6 translation arrest. Gynecologic Oncology. 1992;45(1):84.

3. Plante M, Rubin SC, Wong GY, Federici MG, Finstad CL, Gastl GA. Interleukin‐6 level in serum and ascites as a prognostic factor in patients with epithelial ovarian cancer. Cancer. 1994;73(7):1882-8.

4. Scambia G, Testa U, Benedetti Panici P, Foti E, Martucci R, Gadducci A, et al. Prognostic significance of interleukin 6 serum levels in patients with ovarian cancer. British journal of cancer. 1995;71(2):354-6.

5. Guo Y, Nemeth J, O'Brien C, Susa M, Liu X, Zhang Z, et al. Effects of Siltuximab on the IL-6–Induced Signaling Pathway in Ovarian Cancer. Clinical Cancer Research. 2010;16(23):5759-69.

6. Coward J, Kulbe H, Chakravarty P, Leader D, Vassileva V, Leinster DA, et al. Interleukin-6 as a Therapeutic Target in Human Ovarian CancerIL-6 and Ovarian Cancer. Clinical cancer research. 2011;17(18):6083-96.

7. Anglesio MS, George J, Kulbe H, Friedlander M, Rischin D, Lemech C, et al. IL6-STAT3-HIF signaling and therapeutic response to the angiogenesis inhibitor sunitinib in ovarian clear cell cancer. Clinical cancer research. 2011;17(8):2538-48.

8. Lo C-W, Chen M-W, Hsiao M, Wang S, Chen C-A, Hsiao S-M, et al. IL-6 trans-signaling in formation and progression of malignant ascites in ovarian cancer. Cancer research. 2011;71(2):424-34.

9. Wang Y, Li L, Guo X, Jin X, Sun W, Zhang X, et al. Interleukin-6 signaling regulates anchorage-independent growth, proliferation, adhesion and invasion in human ovarian cancer cells. Cytokine. 2012;59(2):228-36.

10. Cohen S, Bruchim I, Graiver D, Evron Z, Oron-Karni V, Pasmanik-Chor M, et al. Platinum-resistance in ovarian cancer cells is mediated by IL-6 secretion via the increased expression of its target cIAP-2. Journal of Molecular Medicine. 2013;91:357-68.

11. Ataie-Kachoie P, Morris DL, Pourgholami MH. Minocycline suppresses interleukine-6, its receptor system and signaling pathways and impairs migration, invasion and adhesion capacity of ovarian cancer cells: in vitro and in vivo studies. PloS one. 2013;8(4):e60817.

12. Isobe A, Sawada K, Kinose Y, Ohyagi-Hara C, Nakatsuka E, Makino H, et al. Interleukin 6 receptor is an independent prognostic factor and a potential therapeutic target of ovarian cancer. PloS one. 2015;10(2):e0118080.

13. Kim S, Gwak H, Kim HS, Kim B, Dhanasekaran DN, Song YS. Malignant ascites enhances migratory and invasive properties of ovarian cancer cells with membrane bound IL-6R in vitro. Oncotarget. 2016;7(50):83148.

14. Pasquier J, Gosset M, Geyl C, Hoarau-Véchot J, Chevrot A, Pocard M, et al. CCL2/CCL5 secreted by the stroma induce IL-6/PYK2 dependent chemoresistance in ovarian cancer. Molecular Cancer. 2018;17(1):1-14.

15. Wang Y, Zong X, Mitra S, Mitra AK, Matei D, Nephew KP. IL-6 mediates platinum-induced enrichment of ovarian cancer stem cells. JCI insight. 2018;3(23).

16. Yousefi H, Momeny M, Ghaffari SH, Parsanejad N, Poursheikhani A, Javadikooshesh S, et al. IL-6/IL-6R pathway is a therapeutic target in chemoresistant ovarian cancer. Tumori journal. 2019;105(1):84-91.

17. Mehner C, Miller E, Hockla A, Coban M, Weroha SJ, Radisky DC, et al. Targeting an autocrine IL-6–SPINK1 signaling axis to suppress metastatic spread in ovarian clear cell carcinoma. Oncogene. 2020;39(42):6606-18.

18. Ge J, Han T, Shan L, Na J, Li Y, Wang J. Long non-coding RNA THOR promotes ovarian Cancer cells progression via IL-6/STAT3 pathway. Journal of Ovarian Research. 2020;13(1):1-11.

19. Zhang R, Roque DM, Reader J, Lin J. Combined inhibition of IL‑6 and IL‑8 pathways suppresses ovarian cancer cell viability and migration and tumor growth. International Journal of Oncology. 2022;60(5):1-9.

20. Park S-A, Seo YJ, Kim LK, Kim HJ, Yoon KD, Heo T-H. Butein Inhibits Cell Growth by Blocking the IL-6/IL-6Rα Interaction in Human Ovarian Cancer and by Regulation of the IL-6/STAT3/FoxO3a Pathway. International Journal of Molecular Sciences. 2023;24(7):6038.

21. Coward J, Kulbe H, Chakravarty P, Leader D, Vassileva V, Leinster DA, et al. Interleukin-6 as a therapeutic target in human ovarian cancer. Clinical cancer research. 2011;17(18):6083-96.

22. Stone RL, Nick AM, McNeish IA, Balkwill F, Han HD, Bottsford-Miller J, et al. Paraneoplastic thrombocytosis in ovarian cancer. New England Journal of Medicine. 2012;366(7):610-8.

23. Zhang Q, Wang J, Huang F, Yao Y, Xu L. Leptin induces NAFLD progression through infiltrated CD8+ T lymphocytes mediating pyroptotic-like cell death of hepatocytes and macrophages. Digestive and Liver Disease. 2021;53(5):598-605.

24. Angevin E, Tabernero J, Elez E, Cohen SJ, Bahleda R, Van Laethem J-L, et al. A phase I/II, multiple-dose, dose-escalation study of siltuximab, an anti-interleukin-6 monoclonal antibody, in patients with advanced solid tumors. Clinical Cancer Research. 2014;20(8):2192-204.

25. Dijkgraaf E, Santegoets S, Reyners A, Goedemans R, Wouters M, Kenter G, et al. A phase I trial combining carboplatin/doxorubicin with tocilizumab, an anti-IL-6R monoclonal antibody, and interferon-α2b in patients with recurrent epithelial ovarian cancer. Annals of Oncology. 2015;26(10):2141-9.

26. Kim D-K, Oh SY, Kwon H-C, Lee S, Kwon KA, Kim BG, et al. Clinical significances of preoperative serum interleukin-6 and C-reactive protein level in operable gastric cancer. BMC cancer. 2009;9(1):1-9.

27. Ruzzo A, Catalano V, Canestrari E, Giacomini E, Santini D, Tonini G, et al. Genetic modulation of the interleukin 6 (IL-6) system in patients with advanced gastric cancer: a background for an alternative target therapy. BMC cancer. 2014;14(1):1-8.

28. Wang S-W, Sun Y-M. The IL-6/JAK/STAT3 pathway: potential therapeutic strategies in treating colorectal cancer. International journal of oncology. 2014;44(4):1032-40.

29. Ham I-H, Oh HJ, Jin H, Bae CA, Jeon S-M, Choi KS, et al. Targeting interleukin-6 as a strategy to overcome stroma-induced resistance to chemotherapy in gastric cancer. Molecular cancer. 2019;18:1-14.

30. Zhang Y, Xu J, Zhang N, Chen M, Wang H, Zhu D. Targeting the tumour immune microenvironment for cancer therapy in human gastrointestinal malignancies. Cancer letters. 2019;458:123-35.

31. Kinoshita H, Hirata Y, Nakagawa H, Sakamoto K, Hayakawa Y, Takahashi R, et al. Interleukin-6 mediates epithelial–stromal interactions and promotes gastric tumorigenesis. PloS one. 2013;8(4):e60914.

32. Yang H, Xu W. STAT3 promotes peritoneal metastasis of gastric cancer by enhancing mesothelial-mesenchymal transition. Biological Chemistry. 2021;402(6):739-48.

33. Li Y-Y, Hsieh L-L, Tang R-P, Liao S-K, Yeh K-Y. Interleukin-6 (IL-6) released by macrophages induces IL-6 secretion in the human colon cancer HT-29 cell line. Human immunology. 2009;70(3):151-8.

34. Hsu CP, Chen YL, Huang CC, Chou CC, Liu CL, Hung CH, et al. Anti‐interleukin‐6 receptor antibody inhibits the progression in human colon carcinoma cells. European journal of clinical investigation. 2011;41(3):277-84.

35. Ying J, Tsujii M, Kondo J, Hayashi Y, Kato M, Akasaka T, et al. The effectiveness of an anti-human IL-6 receptor monoclonal antibody combined with chemotherapy to target colon cancer stem-like cells. International journal of oncology. 2015;46(4):1551-9.

36. Han J, Xi Q, Meng Q, Liu J, Zhang Y, Han Y, et al. Interleukin-6 promotes tumor progression in colitis‑associated colorectal cancer through HIF-1α regulation. Oncology Letters. 2016;12(6):4665-70.

37. Yin Y, Yao S, Hu Y, Feng Y, Li M, Bian Z, et al. The immune-microenvironment confers chemoresistance of colorectal cancer through macrophage-derived IL6. Clinical Cancer Research. 2017;23(23):7375-87.

38. Zhang X, Hu F, Li G, Li G, Yang X, Liu L, et al. Human colorectal cancer-derived mesenchymal stem cells promote colorectal cancer progression through IL-6/JAK2/STAT3 signaling. Cell death & disease. 2018;9(2):25.

39. Li J, Xu J, Yan X, Jin K, Li W, Zhang R. Targeting interleukin-6 (IL-6) sensitizes anti-PD-L1 treatment in a colorectal cancer preclinical model. Medical science monitor: international medical journal of experimental and clinical research. 2018;24:5501.

40. Li S, Tian J, Zhang H, Zhou S, Wang X, Zhang L, et al. Down-regulating IL-6/GP130 targets improved the anti-tumor effects of 5-fluorouracil in colon cancer. Apoptosis. 2018;23:356-74.

41. Elkazzaz M, Ullah S, Gao T, Shamkh IM, Ahmed A, Wu TX, et al. Inhibition of colorectal cancer targets IL-6, CTLA-4, & B7-2 by Tislelizumab: molecular docking, dynamics, & STRING protein-protein network analysis. Informatics in Medicine Unlocked. 2023;41:101323.

42. Nagasaki T, Hara M, Nakanishi H, Takahashi H, Sato M, Takeyama H. Interleukin-6 released by colon cancer-associated fibroblasts is critical for tumour angiogenesis: Anti-interleukin-6 receptor antibody suppressed angiogenesis and inhibited tumour–stroma interaction. British journal of cancer. 2014;110(2):469-78.

43. Heichler C, Scheibe K, Schmied A, Geppert CI, Schmid B, Wirtz S, et al. STAT3 activation through IL-6/IL-11 in cancer-associated fibroblasts promotes colorectal tumour development and correlates with poor prognosis. Gut. 2020;69(7):1269-82.

44. Zhang H, Wu H, Guan J, Wang L, Ren X, Shi X, et al. Paracrine SDF-1α signaling mediates the effects of PSCs on GEM chemoresistance through an IL-6 autocrine loop in pancreatic cancer cells. Oncotarget. 2015;6(5):3085.

45. Xing H-B, Tong M-T, Wang J, Hu H, Zhai C-Y, Huang C-X, et al. Suppression of IL-6 gene by shRNA augments gemcitabine chemosensitization in pancreatic adenocarcinoma cells. BioMed research international. 2018;2018.

46. Lesina M, Kurkowski MU, Ludes K, Rose-John S, Treiber M, Klöppel G, et al. Stat3/Socs3 activation by IL-6 transsignaling promotes progression of pancreatic intraepithelial neoplasia and development of pancreatic cancer. Cancer cell. 2011;19(4):456-69.

47. Zhang Y, Yan W, Collins MA, Bednar F, Rakshit S, Zetter BR, et al. Interleukin-6 is required for pancreatic cancer progression by promoting MAPK signaling activation and oxidative stress resistance. Cancer research. 2013;73(20):6359-74.

48. Goumas FA, Holmer R, Egberts JH, Gontarewicz A, Heneweer C, Geisen U, et al. Inhibition of IL‐6 signaling significantly reduces primary tumor growth and recurrencies in orthotopic xenograft models of pancreatic cancer. International journal of cancer. 2015;137(5):1035-46.

49. Mace TA, Shakya R, Pitarresi JR, Swanson B, McQuinn CW, Loftus S, et al. IL-6 and PD-L1 antibody blockade combination therapy reduces tumour progression in murine models of pancreatic cancer. Gut. 2018;67(2):320-32.

50. Rupert JE, Narasimhan A, Jengelley DH, Jiang Y, Liu J, Au E, et al. Tumor-derived IL-6 and trans-signaling among tumor, fat, and muscle mediate pancreatic cancer cachexia. Journal of Experimental Medicine. 2021;218(6):e20190450.

51. Ware MB, Phillips M, McQuinn C, Zaidi MY, Knochelmann HM, Greene E, et al. Dual IL-6 and CTLA-4 blockade regresses pancreatic tumors in a T cell–and CXCR3-dependent manner. JCI insight. 2023;8(8).

52. Hurwitz H, Van Cutsem E, Bendell J, Hidalgo M, Li C-P, Salvo MG, et al. Ruxolitinib+ capecitabine in advanced/metastatic pancreatic cancer after disease progression/intolerance to first-line therapy: JANUS 1 and 2 randomized phase III studies. Investigational new drugs. 2018;36:683-95.

53. Ng K, Hendifar A, Starodub A, Chaves J, Yang Y, Koh B, et al. Phase 1 dose-escalation study of momelotinib, a Janus kinase 1/2 inhibitor, combined with gemcitabine and nab-paclitaxel in patients with previously untreated metastatic pancreatic ductal adenocarcinoma. Investigational new drugs. 2019;37:159-65.

54. Bekaii-Saab T, Okusaka T, Goldstein D, Oh D-Y, Ueno M, Ioka T, et al. Napabucasin plus nab-paclitaxel with gemcitabine versus nab-paclitaxel with gemcitabine in previously untreated metastatic pancreatic adenocarcinoma: an adaptive multicentre, randomised, open-label, phase 3, superiority trial. EClinicalMedicine. 2023;58.

55. Chen IM, Johansen JS, Theile S, Madsen K, Dajani O, Lorentzen T, et al. Randomized phase 2 study of nab-paclitaxel and gemcitabine with or without tocilizumab as first-line treatment in patients with advanced pancreatic cancer (PACTO). American Society of Clinical Oncology; 2023.

56. Schmitter D, Lauber B, Fagg B, Stahel RA. Hematopoietic growth factors secreted by seven human pleural mesothelioma cell lines: interleukin‐6 production as a common feature. International journal of cancer. 1992;51(2):296-301.

57. Nakano T, Chahinian A, Shinjo M, Tonomura A, Miyake M, Togawa N, et al. Interleukin 6 and its relationship to clinical parameters in patients with malignant pleural mesothelioma. British journal of cancer. 1998;77(6):907-12.

58. Adachi Y, Aoki C, Yoshio‐Hoshino N, Takayama K, Curiel DT, Nishimoto N. Interleukin‐6 induces both cell growth and VEGF production in malignant mesotheliomas. International journal of cancer. 2006;119(6):1303-11.

59. Adachi Y, Yoshio-Hoshino N, Aoki C, Nishimoto N. VEGF targeting in mesotheliomas using an interleukin-6 signal inhibitor based on adenovirus gene delivery. Anticancer research. 2010;30(6):1947-52.

60. Vlaeminck-Guillem V, Bienvenu J, Isaac S, Grangier B, Golfier F, Passot G, et al. Intraperitoneal cytokine level in patients with peritoneal surface malignancies. A study of the RENAPE (French Network for Rare Peritoneal Malignancies). Annals of surgical oncology. 2013;20:2655-62.

61. Judge S, Thomas P, Govindarajan V, Sharma P, Loggie B. Malignant peritoneal mesothelioma: characterization of the inflammatory response in the tumor microenvironment. Annals of surgical oncology. 2016;23:1496-500.

62. Carr NJ, Bibeau F, Bradley RF, Dartigues P, Feakins RM, Geisinger KR, et al. The histopathological classification, diagnosis and differential diagnosis of mucinous appendiceal neoplasms, appendiceal adenocarcinomas and pseudomyxoma peritonei. Histopathology. 2017;71(6):847-58.

63. Mahečić DH, Berković MC, Zjačić-Rotkvić V, Čačev T, Kapitanović S, Ulamec M. Inflammation-related cytokines and their roles in gastroenteropancreatic neuroendocrine neoplasms. Bosnian journal of basic medical sciences. 2020;20(4):445.
